# Transcriptomic analysis reveals abnormal muscle repair and remodeling in survivors of critical illness with sustained weakness

**DOI:** 10.1038/srep29334

**Published:** 2016-07-14

**Authors:** Christopher J. Walsh, Jane Batt, Margaret S. Herridge, Sunita Mathur, Gary D. Bader, Pingzhao Hu, Claudia C. dos Santos

**Affiliations:** 1 Keenan and Li Ka Shing Knowledge Institute of Saint Michael’s Hospital, Toronto, Ontario, Canada; 2 Institute of Medical Sciences and Department of Medicine, University of Toronto, Toronto, Ontario, Canada; 3 University Health Network, Interdepartmental Division of Critical Care, University of Toronto, Toronto, Ontario, Canada; 4 Department of Physical Therapy, University of Toronto, Toronto, Ontario, Canada; 5 The Donnelly Center, University of Toronto, Toronto, Ontario, Canada; 6 Department of Biochemistry and Medical Genetics, University of Manitoba, Winnipeg, Manitoba; 7 Interdepartmental Division of Critical Care, University of Toronto, Toronto, Ontario, Canada

## Abstract

ICU acquired weakness (ICUAW) is a common complication of critical illness characterized by structural and functional impairment of skeletal muscle. The resulting physical impairment may persist for years after ICU discharge, with few patients regaining functional independence. Elucidating molecular mechanisms underscoring sustained ICUAW is crucial to understanding outcomes linked to different morbidity trajectories as well as for the development of novel therapies. Quadriceps muscle biopsies and functional measures of muscle strength and mass were obtained at 7 days and 6 months post-ICU discharge from a cohort of ICUAW patients. Unsupervised co-expression network analysis of transcriptomic profiles identified discrete modules of co-expressed genes associated with the degree of muscle weakness and atrophy in early and sustained ICUAW. Modules were enriched for genes involved in skeletal muscle regeneration and extracellular matrix deposition. Collagen deposition in persistent ICUAW was confirmed by histochemical stain. Modules were further validated in an independent cohort of critically ill patients with sepsis-induced multi-organ failure and a porcine model of ICUAW, demonstrating disease-associated conservation across species and peripheral muscle type. Our findings provide a pathomolecular basis for sustained ICUAW, implicating aberrant expression of distinct skeletal muscle structural and regenerative genes in early and persistent ICUAW.

Intensive care unit-acquired weakness (ICUAW) describes a spectrum of muscle weakness associated with critical illness that may persist for years after ICU discharge and contributes to significant long-term disability^1^. The first three to six months after critical illness is crucial as many patients have a marked improvement in muscle function before reaching a plateau, resulting in sustained ICUAW^2^,^3^. The biological mechanisms responsible for recovery of muscle strength versus persistence of muscle weakness remain poorly understood. No comprehensive, longitudinal studies concurrently assessing structural, functional and molecular features of ICUAW have been carried out in survivors of critical illness. While molecular data from animal models have been used to infer the molecular pathways of early ICUAW in humans, these models suffer from a number of limitations^4^ and cannot be used to model sustained ICUAW.

A fundamental question regarding the pathomechanism of ICUAW is whether convergent transcriptional changes in response to muscle injury are associated with impaired recovery of muscle mass and strength in ICUAW. We hypothesized that the degree of aberrant expression of genes involved in skeletal muscle regeneration and repair is associated with the extent of muscle atrophy and weakness (muscle phenotypes) in ICUAW^5^,^6^. To test this hypothesis, quadriceps muscle biopsies and functional measures of muscle strength and mass were obtained at 7 days and 6 months post ICU-discharge from a cohort of ICUAW patients enrolled in the RECOVER Program (phase 1: Towards RECOVER)^5^,^6^,^7^ (Supplementary Table 1), a multi-center prospective longitudinal study evaluating functional outcomes in critically ill patients following prolonged mechanical ventilation over a 1 year period, after ICU discharge^5^,^6^. Clinical and functional data for the entire RECOVER and muscle biopsy cohorts are published^8^,^9^.

## Results

**Figure 1:**
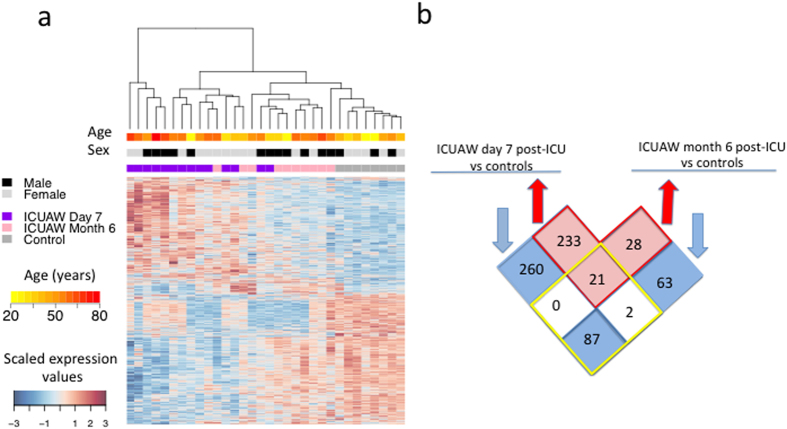
Differentially expressed genes in ICUAW (**a**). Heat map of 695 gene probes differentially expressed between ICUAW at day 7 and ICUAW at month 6 post-ICU versus healthy controls. Differential expression was assessed at a false positive discovery rate (FDR) <0.05 and fold change >1.0. Scaled expression values are color coded according to the legend below the heat map. The top bars indicate patient variables: group (purple, ICUAW day 7; pink, ICUAW month 6; grey, control), age and sex (values are color coded according to respective legend to the right of the heat map). (**b**) Venn diagram of differentially expressed probes in ICUAW day 7 post-ICU (left) and ICUAW 6 months ICU (right). Number of overlapping genes shared between day 7 and month 6 are shown within the four squares within the yellow diamond; number of genes exclusively differentially expressed in ICUAW day 7 (left) or ICUAW month 6 post-ICU (right) are shown in the 4 squares outside the yellow diamond.

We analyzed 32 *vastus lateralis* muscle tissue samples from 14 ICUAW patients (14 biopsies at day 7 and 10 follow-up biopsies at month 6 post-ICU discharge) and 8 healthy controls (Supplementary Table 1) using Illumina microarrays. For the probes that passed quality control their expression was adjusted for age, sex, and correlation between samples from the same patient (methods). A total of 695 genes were found to be differentially expressed between 14 ICUAW day 7 post-ICU, 10 ICUAW month 6 post-ICU, and 8 control samples, using a cut-off false discovery rate (FDR) <5% (Supplementary Table 2). Unsupervised hierarchical clustering showed skeletal muscle profiles from ICUAW patients clustered independent of age and sex (Fig. 1a). Samples also clustered independently from severity of critical illness upon ICU admission and pre-morbid status (data not included).

A total of 347 genes were under-expressed (downregulated) day 7 post-ICU compared to controls. Eighty-seven of these were also downregulated in month 6 post-ICU samples (Fig. 1b). Twenty-one of 256 genes upregulated at day 7 were also upregulated at month 6 post-ICU discharge versus controls. Genes significantly downregulated in ICUAW day 7 post-ICU compared to controls showed enrichment for the Gene Ontology (GO) and Human Phenotype Ontology (HPO) terms that underscore the role of bioenergy metabolism and mitochondrial dysfunction in acute muscle injury including mitochondrial inner membrane (*P*_hypergeometric_ = 8.50 × 10^−11^) and acidosis (*P*_hypergeometric_ = 0.0129) (Supplementary Table 3), compatible with findings from animal models of ICUAW and cohorts of sepsis with multiple organ dysfunction syndrome (MODS)^10^,^11^. No significant enrichment of GO terms was detected for upregulated genes at day 7 post-ICU or up- (*N* = 50) or downregulated (*N* = 152) genes at month 6 post-ICU compared to controls. Although biopsy samples from patients at day 7 post-ICU discharge clustered separately from those samples collected 6 months post-ICU discharge this single-gene approach failed to identify clinically relevant molecular correlates.

Since co-expression network analysis has been found to uncover clinically relevant modules (interconnected groups of co-expressed genes) in cross-sectional and time-series studies of complex diseases^12^,^13^ we used weighted gene co-expression network analysis (WGCNA) to detect co-expression modules that correlate with clinical muscle phenotypes. Accordingly, a co-expression network based on age and sex adjusted expression data was built using the entire dataset, containing all ICUAW and control samples (32 muscle transcriptomes), identifying 17 co-expression modules, labeled numerically by module size, with module 1 (M1) indicating the largest module (Supplementary Table 4). Module expression for each sample was summarized by the first principle component of its expression (module eigengene [ME]).

We defined ICUAW-relevant modules as those having ME associated with case-control status for at least one clinical variable. Modules with differential expression of ME at day 7 post-ICU versus controls and month 6 post-ICU versus controls were termed “early ICUAW-relevant” and “sustained ICUAW-relevant” modules, respectively. Differences in ME expression were tested using a linear mixed effects model to account for correlation between patient samples. Clinical variables tested for correlation with ME included the motor subscore of the Functional Independence Measure (FIM), global muscle strength measured by MRC sum score (MRCSS) and quadriceps cross sectional area (CSA), expressed as a percentage of published age and sex matched norms, each measured at 7 days and 6 months, and muscle strength determined by quadriceps peak torque (% predicted)^14^ at month 6 post-ICU discharge (Supplementary Figure 1 and Supplementary Table 5).

**Figure 2:**
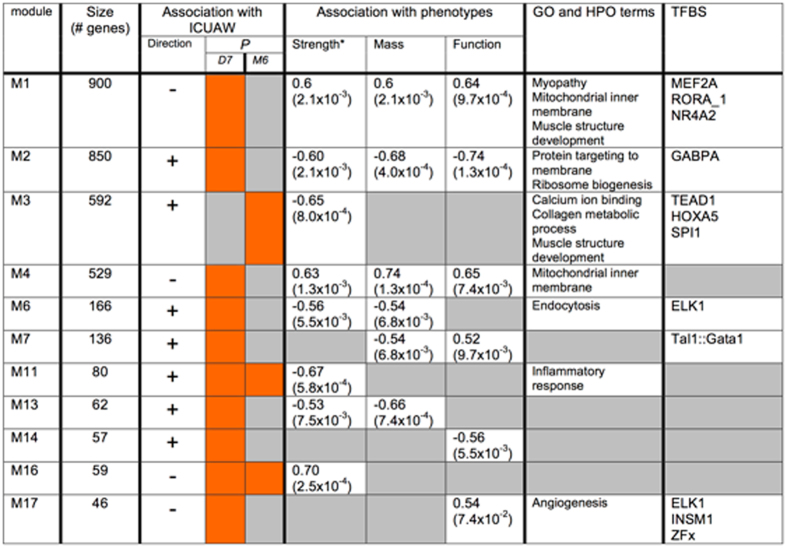
Weighted gene correlation network identifies eleven ICUAW-associated modules. Association with ICUAW for each module, represented by the module eigenegene (first principal component). Direction of differential expression: A positive sign (+) indicates upregulation, and negative sign (−) downregulation of ICUAW samples compared to controls. Ten modules are significantly associated with ICUAW day 7 post-ICU at FDR <0.05 (Differential expression [DE] *P*-value: red boxes in the left-hand column indicate significant DE in ICUAW day 7 post-ICU,), and three modules are associated with ICUAW month 6 post-ICU (red boxes in right column). Grey boxes in left and right columns indicate no significant DE in ICUAW at day 7 and month 6 post-ICU discharge, respectively. Module phenotype correlations (*p*-value in brackets) for muscle strength (based on highest absolute value of either MRCSS or peak torque), muscle mass, and physical function (FIM score). Significantly enriched gene ontology or human phenotype ontology terms, based on FDR <0.05 (representative examples listed). Significantly over-represented transcription factor binding sites (TFBS) based on *P*-value < 9.1 × 10^−4^ (three most significant TFBS above cutoff shown per module).

Eleven of 17 modules met criteria for ICUAW-relevant modules composed of eight early, one sustained, and two early and sustained ICUAW-relevant modules. Seven of the 11 disease-relevant modules, were functionally enriched for GO terms (Fig. 2 and Supplementary Table 6) including modules M1 and M4, each enriched for the GO term mitochondrial inner membrane and significantly downregulated at day 7 post-ICU (*P* = 5.8 × 10^−5^, 3.3 × 10^−5^ respectively), concordant with our single-gene differential expression analysis. Unlike single-gene analysis, we detected functional enrichment in an additional five differentially expressed modules, corroborating the increased power of co-expression network analysis, recognized in other complex diseases, to detect disease-relevant transcriptional changes^12^.

We further hypothesized that genes within an ICUAW-associated module were co-regulated by shared transcription factor binding sites (TFBS)^13^. Analysis of conserved TFBS showed significant over-representation of at least one TFBS in six of 11 modules (M1, M2, M3, M6, M7, M17), many of which are known to be associated with muscle related pathways (as expanded upon below). This suggests that shared TFBS may be co-regulating gene expression in ICUAW-associated modules (Fig. 2 and Supplementary Table 7).

**Figure 3:**
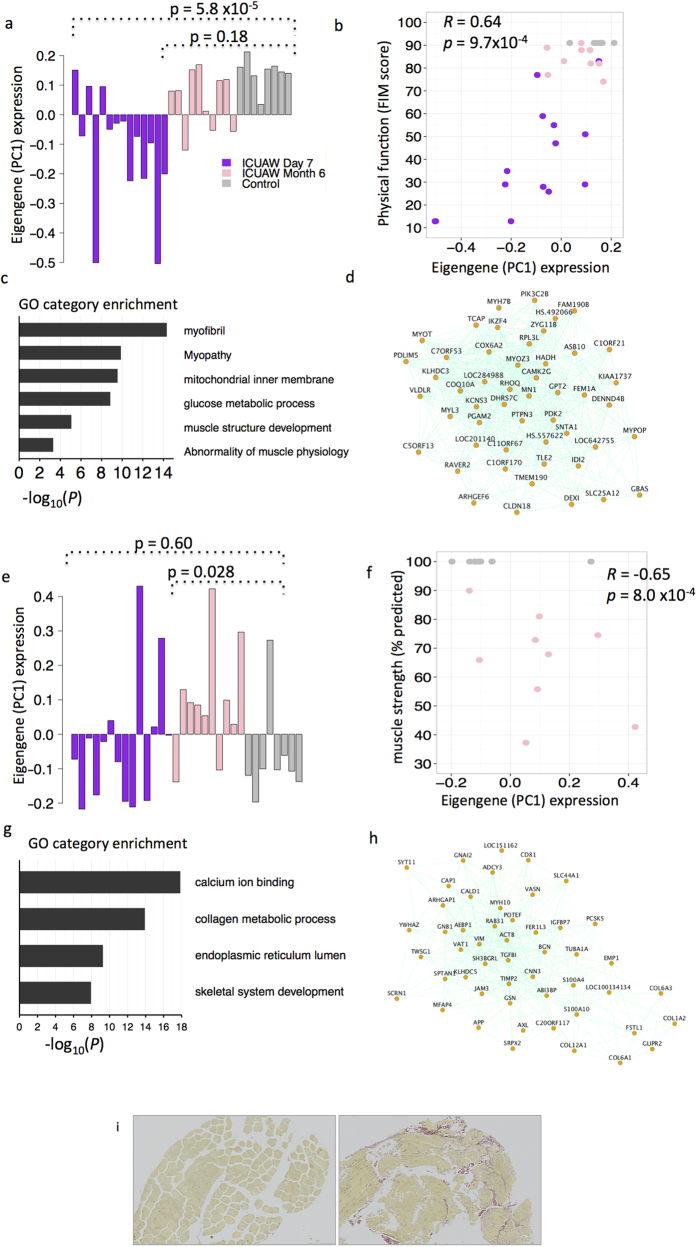
Module 1 and 3 are associated with ICUAW at day 7 and month 6 post-ICU discharge, respectively. (**a,e**) Module eigengene values (y-axis) across samples (x-axis), Purple, ICUAW day 7 post ICU; pink, ICUAW month 6 post ICU; grey, controls. *P*-values of linear mixed effects regression with age and sex as fixed effects. (**b,f**) Module eigengene values (y-axis) vs. clinical measurements (x-axis). Correlation *R*-values calculated using Turkey’s Biweight correlation, *p*-values adjusted for multiple comparisons. (**c,g**) Relevant gene ontology categories enriched in the M1 and M3 modules. (**d,h**) Top 50 most highly connected genes in the M1 and M3 modules. **i** Low magnification (10x) representative photomicrographs of *vastus lateralis* muscle cross sections from a healthy control (left panel) and a sustained ICUAW patient at month 6 (right panel) stained with Picro-Sirius Red Stain. Muscle stains yellow while collagen stains red.

Among the 11 ICUAW-relevant modules, M1 and M3 were found to have particular relevance to muscle biology. The largest early ICUAW-relevant module, M1 (*N* = 900 genes) was downregulated at day 7 post-ICU (*P* = 5.77 × 10^−5^), whereas no difference in expression was detected at month 6 post-ICU compared to controls (Fig. 2). Module eigengene expression correlated to quadriceps CSA (% of age/sex matched population based norm) (*R* = 0.60, p = 2.1 × 10^−3^) and global muscle strength measured by MRCSS (*R* = 0.64, p = 9.7 × 10^−4^). Genes in this module were significantly enriched for multiple GO terms relating to mitochondrial function, bioenergy metabolism, and muscle structure development (Fig. 3a–d and Supplementary Figure 2a). We found significant overrepresentation of multiple TFBS including the myocyte enhancer factor 2A (*MEF2A*) binding site (*P*_hypergeometric_ = 1.43 × 10^−9^). MEF2A has been shown to be upregulated during muscle regeneration^15^ and is required for adult early myogenic (myoblast) differentiation and regeneration in response to injury^16^. Module M2 (N = 850 genes) was upregulated at day 7 post-ICU and inversely correlated with quadriceps CSA (R = −0.68, p = 4.0 × 10^−4^) and global muscle strength (*R* = −0.60, p = 2.1 × 10^−3^). The module was enriched for genes related to ribosome biogenesis (p = 9.62 × 10^−13^) and genes targeting to the membrane (p = 3.01 × 10^−5^). The GA-binding protein alpha (GABPA) TFBS was found to be significantly overrepresented in M2 (*P*_hypergeometric_ = 1.23 × 10^−4^). GABPA has been shown to regulate the expression of synaptic genes at the neuromuscular junction^17^.

The largest sustained ICUAW-relevant module, M3 (*N* = 592 genes) had no difference in ME expression at day 7 post-ICU, but was upregulated at 6 months post-ICU (*P* = 0.028). Module eigengene expression was inversely proportional to muscle strength as determined by quadriceps peak torque at 6 months (*R* = −0.65, P = 8.0 × 10^−4^). The module was enriched for multiple terms relating to wound healing and repair such as extracellular membrane deposition, calcium handling (contractile function), and muscle structure development, suggesting activation of regenerative pathways (Fig. 3e–h, Supplementary Figure 2b). The most significantly overrepresented TFBS in M3, TEAD1 (*P*_*hypergeometric*_ = 1.8 × 10 × 10^−21^), has been shown to be a mediator of skeletal muscle development^18^,^19^. The skeletal muscle development genes enriched in M3 were distinct from those in M1, indicating specific temporal alterations in skeletal muscle regenerative expression profiles in early and sustained ICUAW. Proof of concept for the *in silico* prediction of increased extracellular membrane deposition was corroborated by staining for collagen in a representative sustained ICUAW biopsy sample compared to healthy control (Fig. 3i).

**Figure 4:**
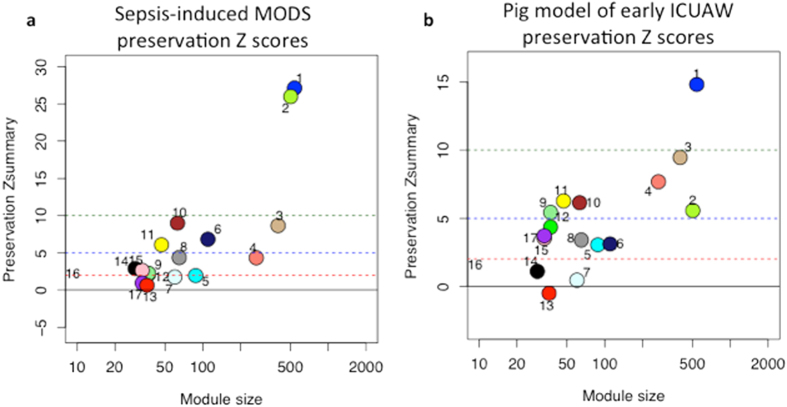
Modules M1 and M3 from human ICUAW patients are preserved in two independent data sets. Summary preservation statistic based on permutation testing Z summary score, using the human ICUAW patients as a reference. The y axis displays the Z score for each module in (**a**) human sepsis-induced multiple organ dysfunction and (**b**). Pig model of ICUAW; numbers beside each module (colored circle) indicate the corresponding module in the reference dataset. The x-axis indicates the number of genes in the module. Z scores of less than 2 (bottom red line) implies no evidence for module preservation, while scores exceeding 5 (green line) and exceeding 10 implies moderate and strong evidence for module preservation, respectively.

We externally validated the robustness of our findings by determining whether the modules detected in our ICUAW dataset were preserved in an experimental pig (*Sus scrofa*) model of sepsis-induced early ICUAW and a human cohort of sepsis-induced MODS biopsied at an average of 7 days after admission to ICU. Previously annotated homologues were used to detect genes shared between pig and human in our dataset^20^, resulting in a network of 5344 genes consisting of the intersection all three datasets. We then tested for preservation of module structure in the validation datasets using a permutation based composite Z statistic, *Z*_summary_ to assess the significance of the observed preservation statistics, where *Z*-values >10 imply significant preservation of module structure (on line methods). Module M1 was significantly preserved in both the cohort of sepsis-induced MODS (*Z* = 27.1, Fig. 4a) and the pig model of ICUAW (*Z* = 14.8, Fig. 4b). Module M3 was modestly preserved in the MODS cohort (*Z* = 8.6, Fig. 4a) and the pig model of ICUAW (*Z* = 9.5, Fig. 4b).

To assess differences in module expression between ICUAW and controls in the validation cohorts we tested the association between the ME and disease status using linear mixed effects regression analysis. Concordant with the findings from our cohort, early ICUAW-relevant module M1 was downregulated in both sepsis-induced MODS versus controls (p = 4.52 × 10^−6^) and the porcine ICUAW model at day 5 versus pre-sepsis day 1 (*p* = 2.22 × 10^−5^) (Supplementary Table 8) showing preservation of module structure and directionality of ME expression change.

## Discussion

We have performed the first longitudinal study of ICUAW to integrate clinical measurements of muscle mass and strength with transcriptomic profiles of skeletal muscle. We hypothesized that the degree of aberrant expression of genes involved in skeletal muscle regeneration and repair is associated with the extent of muscle atrophy and weakness (muscle phenotypes) in ICUAW. Using differential expression (DE) analysis we found distinct expression signatures between early and persistent ICUAW compared to controls. Functional analysis of the differentially expressed genes in early ICUAW found downregulation of mitochondrial genes suggesting bioenergetic failure, in keeping with previous studies^21^.

However, DE analysis of genes does not account for variability of muscle phenotypes within our ICUAW cohort. Therefore we applied co-expression analysis to detect groups of genes whose expression correlates with muscle phenotypes and differentiates ICUAW from healthy controls (DE modules), termed ICUAW-relevant modules. We found one early and one sustained ICUAW relevant module that were enriched for skeletal muscle regeneration genes. One sustained ICUAW-relevant module upregulated for extracellular matrix genes was inversely correlated to muscle strength, suggesting that aberrant muscle repair may hinder force-generation in sustained ICUAW. We verified the presence of collagen deposition in samples of sustained ICUAW using histochemical stains. Using two publically available transcriptomic datasets of early ICUAW we externally validated the module downregulated in early ICUAW and enriched in genes for muscle regeneration and the mitochondria. While we found moderate preservation of the largest sustained ICUAW-relevant module compared to the early ICUAW datasets, there are currently no other datasets of sustained ICUAW available for comparison.

Co-expression analysis has been previously shown to detect enrichment of prognostic relevant genes^22^ and transcription factor binding sites (TFBS)^13^. Indeed, we detected significant overrepresentation of TF binding sites (TFBS) in the majority of ICUAW-relevant modules, suggesting that co-expression of genes in ICUAW-relevant modules are attributable to co-regulation by common TFs. Strikingly, in modules related to muscle regeneration, the most enriched TFBS have experimental evidence supporting their roles in muscle development and differentiation. MEF2A, a TF critical for skeletal muscle regeneration^16^,^23^, was enriched in an early ICUAW-relevant module (M1) downregulated in early ICUAW, suggesting impaired binding of MEF2A to its promoter is associated with the degree of weakness and atrophy in early ICUAW.

TEAD1 binding sites, the muscle CAT (MCAT) elements, were enriched in the sustained ICUAW module (M3) upregulated at 6 months post-ICU. TEAD-binding sites are found in the promotors of genes involved in terminal differentiation and co-activated by the Hippo pathway transduction^19^,^24^,^25^ RUNX1 and NFATC2, also enriched for TFBS in Module 3, have been associated with response to muscle damage^26^,^27^. The calcium-regulated transcription factor NFATC2 is activated only in newly formed myotubes and its gene targets play a key role in myoblast fusion and myotube growth^28^. The RUNX1-mediated transcriptional program regulates muscle-specific genes and structural proteins, controlling the balance of proliferation and differentiation in myoblasts during muscle regeneration^26^. The corroboration of our *in silico* analysis with *in vivo* experimental studies of muscle regeneration therefore serves to further validate our findings. The inverse correlation of muscle strength with M3 gene expression and enrichment of these genes for the extracellular matrix and muscle development genes may denote aberrant muscle regeneration programs resulting in impaired muscle structure and function in sustained ICUAW.

In summary, application of WGCNA, a robust and unbiased systems-level method for gene network analysis has significantly extended our understanding of ICUAW-specific transcriptional alterations compared to individual gene expression analysis. Our study identified distinct transcriptional alterations in the early and sustained phases of ICUAW. The strength of our study, the largest transcriptomic analysis of ICUAW, was the integration of comprehensive clinical measurements with gene network analysis. We have identified striking correlations between module expression and clinical measures of muscle mass and function, suggesting an association between disease-perturbed networks and phenotypic changes. Importantly, the relationships between module expression and clinical traits identified here do not imply causation but they provide a foundation for future ongoing research. Among the 11 ICUAW-relevant modules, 4 did not have any functional enrichment making biological interpretation of these modules less clear. Increased sensitivity of gene module functional enrichment in future analysis may be gained with increasing sample size, use of RNA sequencing, and transcriptomic analysis of single cells. Our analysis showed biologically meaningful enrichment in the majority of gene modules and provides novel insights regarding potential molecular mechanisms of sustained ICUAW. An important theme that emerged from our analysis was temporally distinct alterations of skeletal muscle regenerative genes in early and sustained ICUAW. The robustness of these findings was further supported by preservation of early ICUAW gene networks in other datasets of ICUAW. Thus we have established a comprehensive gene network-based framework to study candidate genes associated with muscle weakness in early and sustained ICUAW. We anticipate that these findings may provide avenues for development of therapies to ameliorate ICUAW in the future.

## Methods

Patient selection and outcome measures are described in Supplementary methods. Written informed consent was obtained from all participants or their surrogate decision makers and participants were re-consented when capacity was regained. The study protocol was approved by the University Health Network Research Ethics Board and St. Michael’s Hospital Research Ethics Board. All methods were performed in accordance with the relevant guidelines and regulations within the study protocol.

### Microarray samples and Quality control

#### Muscle sample collection

Percutaneous muscle biopsy of the *vastus lateralis* was performed at day 7 post-ICU discharge (*n* = 14) and 6 months post-ICU admission (*n* = 10) (Supplementary methods). Healthy muscle biopsy samples (controls) were obtained from previously banked specimens collected from consenting individuals (*n* = 8). *RNA extraction and microarray hybridization.* RNA was processed, amplified, and labeled as previously described^29^. RNA samples included in the expression analysis had high RNA quality (median RNA integrity number [RIN] of 8.5). Expression profiles were obtained using IlluminaHT-12 V4 microarrays (1 microarray per sample). All microarray data are deposited in GEO under accession number GSE78929. *Microarray data analysis.* Microararray data analysis was performed using the R software and Bioconductor packages. Raw expression data were background corrected, quantile normalized and log_2_ transformed using the *neqc* function in the *limma* package. Data quality control included high inter-array correlation (Pearson correlation coefficients >0.85) and detection of outlier arrays based on mean inter-array correlation and hierarchical clustering. All samples fulfilled data quality control criteria. Probes listed as “No match” or “Bad” using the *illuminHumanv4* package were removed, resulting in 34,476 high-quality probes. Robustly expressed probes were defined as those with detection *P* value < 0.05 for at least half of the samples in the data set and standard deviation of probe expression >0.25 (11,482 probes, corresponding to 9869 unique genes).

Muscle staining and Independent validation datasets are described in Supplementary methods.

### Single-gene differential expression analysis

Differential expression of all robustly expressed probes (see above) in ICUAW day 7 and month 6 post-ICU and control samples was assessed in *limma* as gene-wise linear models adjusted for patient age and sex and for consensus correlation between patient samples using the duplicateCorrelation function^30^. Moderated *F*-statistics combined *t*-statistics for all three pair-wise comparisons (contrasts) into an overall test of significance for each gene used. The decideTests function with “global” setting performed error rate control across multiple contrasts and genes simultaneously with pre-specific significant threshold Benjamini Hochberg FDR <5% and absolute values of fold change >1.0. Diagnostic plot of the linear model fit (gene-wise residual standard deviations against average log-expression) were examined and no variance trends were identified. One gene with differential expression in both up and downregulated probes at month 6 post-ICU was removed.

### Co-expression network analysis

Signed hybrid co-expression networks were detected using the WGCNA package in R. We chose signed networks as they have been shown to detect modules with more significant enrichment of functional classifications^31^. We used a linear mixed model (LMM) to correct the gene expression for age and sex effects (fixed effects) with random intercept terms to account for correlation among samples from the same patient and for potential differences in age- or sex-related expression effects between ICUAW and controls. Residual values from the LMM were used for input in WGCNA and the remainder of the analysis^32^. Pairwise Tukey’s Biweight correlations for the set of genes were calculated using the bicor function as this correlation method is more robust than Pearson correlation and often more powerful than Spearman correlation^33^. Adjacency transformation was calculated by raising the correlation matrix to the power of 6, which was chosen using the scale-free topology criterion^34^. For each pair of probes the topological overlap measure was calculated based on the adjacency matrix and the topological overlap dissimilarity measure was used as input for average linkage hierarchical clustering. The Dynamic Hybrid tree cutting algorithm was used to cut branches off the dendrogram because it produces robustly defined modules^35^. To obtain moderately large and distinct modules we set the minimum module size to 50 probes and the minimum height for merging modules set at 0.2 (default parameter). Each module was summarized by its first principle component of the scaled module age and sex corrected (residual) expression values (module *eigengene*). Probes were assigned to a module if they had high module membership (correlation between gene expression and module *eigengene* >0.5). Modules were numerically labeled by module size, with M1 indicating the largest module.

We then tested the association of each module eigengene with disease status (ICUAW at day 7 and ICUAW month 6 vs control) using a mixed effects model with random intercept term to account for correlation among the eigengenes from the same patient with pre-specific significance threshold FDR <5%. P-values were adjusted for multiple tests using the *p.adjust* function (Benjamini Hochberg) in the stats package in R. The co-expression analysis on the 11,482 age and sex corrected probes identified 17 modules, however 6846 probes that did not fulfill these criteria were assigned to the predefined module M0 (designating non-module genes). The module membership of all 11, 482 probes are shown in Supplement Table 4. The association of each module eigengene to disease status is shown in Supplementary Table 5.

Module visualization: for each module the topological overlap measure (kME) was calculated and used to rank genes within the module. The top 50 ranked genes for each module were visualized using Cytoscape.

#### Module-clinical variable relationships

Tukey’s biweight correlation were calculated between continuous clinical variables and module eigengene values. Significant correlation to clinical variables was empirically defined as R ≥0.5 and adjusted p-value < 0.05 (Supplementary Figure 2).

### Gene ontology and Human phenotype ontology analysis

Functional enrichment in Gene Ontology (GO) and Human Phenotype Ontology (HPO) was performed using the gProfiler tool in R^36^. The statistical significance threshold level for GO and HPO enrichment was Benjamini and Hochberg corrected p < 0.05 and a minimum of 10 genes detected. The background list for the enrichment analysis included all genes represented on the Illumina Human HT-12 v4 array with a detection *P* value < 0.05 in at least three samples. Gene set visualization using enrichment map is described in Supplementary methods.

### Transcription factor binding site analysis

oPOSSUM, version 3.0^37^ (http://opossum.cisreg.ca/oPOSSUM3), was used to detect enrichment of human transcription factor binding sites (TFBSs) in the 5,000bp upstream and downstream sequence of input genes (single site analysis; cutoffs were *z* score of ≥10, Fisher exact test score [negative natural logarithm of the hypergeometric *p*-value] of ≥7, default values in oPOSSUM based on empirical studies, and conservation cut-off 0.6). Results were ranked by Fisher exact score and the top three highest scoring TFBS above cutoffs were selected (Supplementary Table 7).

### Preservation analysis

To make the data from different microarray platforms comparable, we converted the probe-level measurements from the validation datasets and our dataset of ICUAW samples to genes-level measurements using *CollapseRows* function in WGCNA using the “MaxMean” setting. The two validation sets contained 5344 of the 9683 (55.2%) of genes used in our co-expression analysis. The 5344 genes in the ICUAW dataset (reference dataset) were mapped to their corresponding module labels from the co-expression analysis using the original 11,482 gene probe dataset. The module membership from the reference data is applied to each validation dataset tested. We applied the module preservation statistic Z_summary_^38^ in the *modulePreservation* function in WGCNA. The Z_summary_ statistic integrates the overlap in module membership with the connectivity (sum of connections) and density (mean connectivity) patterns of the each module. The recommended significance thresholds, Z_summary_ < 2 implies no evidence for module preservation, 2 < Z_summary_ < 10 implies weak to moderate evidence, and Z_summary_ > 10 implies strong evidence for module preservation.

## Additional Information

**How to cite this article**: Walsh, C. J. *et al.* Transcriptomic analysis reveals abnormal muscle repair and remodeling in survivors of critical illness with sustained weakness. *Sci. Rep.* **6**, 29334; doi: 10.1038/srep29334 (2016).

## Supplementary Material

Supplementary Table

Supplementary Information
