# Supplementary material for: Transcriptomic analysis reveals abnormal muscle repair and remodeling in survivors of critical illness with sustained weakness
Source: Sci Rep. 2016 Jul 14;6:29334. doi: 10.1038/srep29334 (PMC4944143; doi:10.1038/srep29334)
Supplement: Supplementary Information [file srep29334-s2.pdf]

Transcriptomic analysis reveals abnormal repair and remodeling in survivors of critical illness with sustained muscle weakness

Christopher J Walsh MD FRCPC[1,2], Jane Batt MD FRCPC PhD[1,2], Margaret S. Herridge MD FRCPC MPH[3], Sunita Mathur PhD[4], Gary D. Bader PhD[5], Pingzhao Hu, PhD[6], and Claudia C. dos Santos MD FRCPC MSc[1,2]

1. Keenan and Li Ka Shing Knowledge Institute of Saint Michael's Hospital, Toronto, Ontario, M5B 1W8, Canada.
2. Institute of Medical Sciences and Department of Medicine, University of Toronto, Toronto, Ontario, Canada.
3. University Health Network, Interdepartmental Division of Critical Care, University of Toronto, Toronto, Ontario, Canada
4. Department of Physical Therapy, University of Toronto, Toronto, Ontario, Canada
5. The Donnelly Center, University of Toronto, Toronto, Ontario, Canada
6. Department of Biochemistry and Medical Genetics, University of Manitoba, Winnipeg, Manitoba

*Correspondence and reprint requests to:
C. C. dos Santos, MSc, MD.
Clinician-Scientist, Associate Professor of Medicine,
Keenan Research Centre for Biomedical Science of St Michael's Hospital and Interdepartmental Division of Critical Care, University of Toronto
St Michaels Hospital, 30 Bond Street, Room 4-008, Toronto, ON, M5B 1WB, CA.
Tel: (+1416)-864-6060 (3198)
Fax: (+1416)-864-6013
E-mail: dosSantosC@smh.ca

List of supplementary data

PDF (a single file)

1. Supplementary Methods
2. Supplementary Figure 1.
Heatmap of correlations between co-expression module eigengenes (rows) and quantitative clinical traits (columns). The clinical traits (from left to right) are FIM motor subscore, Functional Independence Measure (motor subset score); Quads mass, quadriceps cross sectional area expressed as a percentage of age and sex matched norms determined by computerized tomography; APACHE2 score, a severity of disease classification system; MRCSS, Medical Research Council sum score (a sum score of global muscle strength); Quads strength, quadriceps strength determined by quadriceps peak torque (% predicted); RIN, RNA integrity number. Scale bar (right) indicates the range of possible correlations from positive (red, 1) to negative (green, -1).
3. Supplementary Figure 2.
Enrichment Map results of the gene set functional enrichment analysis for (a) module 1 and (b) module 3. A node in the Enrichment Map

represents a gene set. Node size represents gene set size. Node color, blue or red, indicates whether gene sets are down- or up-regulated compared to controls, respectively.

4. Supplementary Table 1
   Patient demographic and clinical data
5. Supplementary Table 2
   Differentially expressed genes (N = 744 unique probes corresponding to 695 genes). LIMMA global analysis of differentially expressed genes between ICUAW and controls for both day 7 post-ICU and month 6 post-ICU at false discovery rate (FDR) 5% level was performed using data adjusted for age, sex, and correlation between patient samples.
6. Supplementary Table 3
   Enrichment analysis of 347 genes differentially expressed (downregulated in ICUAW post-ICU day 7 versus controls) at FDR < 0.05.
7. Supplementary Table 5
   Association of co-expression modules with disease status
8. Supplementary Table 6
   6a . Module 1 and enrichment of Gene Ontology and Human Phenotype Ontology with FDR < 0.05. 6b. Module 2 and enrichment of Gene Ontology and Human Phenotype Ontology with FDR < 0.05. 6c. Module 3 and enrichment of Gene Ontology and Human Phenotype Ontology with FDR < 0.05. 6d. Module 4 and enrichment of Gene Ontology and Human Phenotype Ontology with FDR < 0.05. 6e. Module 11 and enrichment of Gene Ontology and Human Phenotype Ontology with FDR < 0.05. 6f. Module 17 and enrichment of Gene Ontology and Human Phenotype Ontology with FDR < 0.05.
9. Supplementary Table 7
   7a. Module 1 and transcription factor binding site overrepresentation analysis with Fisher score >= 7 and Z-score >= 10. 7b Module 2 and transcription factor binding site overrepresentation analysis with Fisher score >= 7 and Z-score >= 10. 7c Module 3 and transcription factor binding site overrepresentation analysis with Fisher score >= 7 and Z-score >= 10. 7d Module 6 and transcription factor binding site overrepresentation analysis with Fisher score >= 7 and Z-score >= 10. 7e Module 7 and transcription factor binding site overrepresentation analysis with Fisher score >= 7 and Z-score >= 10. 7f Module 17 and transcription factor binding site overrepresentation analysis with Fisher score >= 7 and Z-score >= 10.
10. Supplementary Table 8
   8a. Module conservation between modules constructed for the human sepsis with multi-organ dysfunction validation dataset compared with reference (human ICUAW). 8b. Module conservation between modules constructed for the porcine model of ICUAW compared with reference (human ICUAW). 8c. Association of module eigengene (ME) with disease status; human sepsis with multi-organ dysfunction vs controls 8d. Association of ME with disease status; porcine model of ICUAW day 5 sepsis vs. day 1 control.

Excel

1. Supplementary Table 4

   Gene and kME in different modules. Module eigengene connectivity (kME), a module membership measure, for all unique Illumina probes included in the WGCNA analysis data set (N= 11,482)

Supplemental Methods:

Patient selection: Patients were enrolled from a multi-center nested prospective study (Towards RECOVER)[1,2]. Patients were included if mechanically ventilated for a minimum of 1 week and were fully ambulatory and independent with activities of daily living prior to ICU admission. Exclusion criteria included any of the following: important current or pre-existing neurological injury that would preclude cognitive testing; a formal diagnosis of neuromuscular disease; non-ambulatory prior to ICU; anticipated death or withdrawal of life sustaining treatment within 48 hours; history of psychiatric illness or significant cognitive impairment with documented hospital admission; not fluent in English or French; living greater than 300 km from referral centre; physician refusal; patient or SDM (substitute decision maker) consent refusal; and no available next of kin or SDM available (if patient unable to provide consent). Additional exclusion criteria for this nested study included important current neurological injury that would preclude motor testing; known HIV, Hepatitis B or Hepatitis C infection; therapeutic anticoagulation and/or active cancer undergoing treatment.

Written informed consent was obtained from all participants or their surrogate decision makers and participants were re-consented when capacity was regained. The study protocol was approved by the University Health Network Research Ethics Board and St. Michael's Hospital Research Ethics Board. All methods were performed in accordance with the relevant guidelines and regulations within the study protocol. Banked muscle biopsy specimens previously collected from consenting healthy individuals were used as controls.

All controls had been screened by interview and self-reported the absence of malignancy, significant cardiac, pulmonary, hepatic, renal or endocrine disease.

Outcome measures of physical function, strength and mass: Physical functional capacity was measured using the motor component of the Functional Independence Measure questionnaire (FIM score) [3]. Functional Independent Measures questionnaire (FIM) provides a numerical score for cognitive and motor function that has been validated and standardized across diverse patient populations. A higher FIM score (scale 18-126) connotes better function in both domains. The motor subscore is based on the individual's ability to perform their activities of daily living (ADLs; toileting, dressing, walking, climbing stairs, eating). A healthy individual with complete and unassisted independence of ADL would achieve a maximal FIM motor subscore of 91. The measure is independent of an individual's gender or age.

Global muscle strength was assessed by the Medical Research Council Sum Score (MRCSS) [4]. Manual assessment of muscle strength was performed by a physiotherapist, grading muscle strength from 0 to 5, as established by the Medical Research Council. The higher the score, the stronger the muscle group, with 5 representing what is deemed by the assessor to be normal strength, taking into account the individual's sex and age. The MRC sum score is calculated by summing the total score for bilateral shoulder abduction, elbow flexion, wrist extension, hip flexion, knee extension, and ankle dorsiflexion. The score for an individual with normal strength is 60.

The midthigh *quadriceps femoris* muscle cross-sectional area (CSA) was determined as previously described [5]. Briefly, computed tomography imaging of

the right thigh, halfway between the pubic symphysis and the inferior condyle of the femur, using a Light Speed QXi 4 slice helical scanner (General Electric, Milwaukee, WI), was performed with the subject in the supine position. Each image was 10 to 20 mm thick, and the muscle identified as tissue with a density of 40 to 100 Hounsfield units. Images were analyzed and the CSA of thigh muscle determined by a single radiologist, blinded as to the categorization of each test subject. Quadriceps was then manually traced using ImageJ software (version 1.42q, NIH, USA) by a single investigator blinded to participant categorization and the CSA calculated. The cross-sectional area of the muscle at the midsection of the thigh determined by computerized tomography (CT) was compared to age and sex matched publication based norms [6,7,8,9,10] and muscle mass for study subjects is expressed as a percent of the age and sex matched norms.

Quadriceps isometric peak torque (strength) was measured from maximal voluntary contractions of the knee extensors. The patient was seated on the Biodex dynamometer (Biodex System 4), with the hip at 85 degrees and knee at 60 degrees of flexion. The patient performed five maximal voluntary contractions with one minute rests, according to a previously described protocol [11]. Peak torque was recorded in Newton-meters (Nm). Patients' peak torque was reported in absolute terms and as percentage of the predicted normal, as previously described [12]. Predicted Quadriceps Force in Nm = - (2.21 x age) + (55.9 x gender [female = 0, male = 1]) + (1.78 x Body weight) + 124.

All testing was conducted at 7 days and 6 months post-ICU discharge, with the exception of the Biodex measures, which were conducted solely at 6 months. For controls, the values for FIM motor subscore, MRCSS, percent predicted quadriceps isometric peak strength, and percentage of normal

quadriceps CSA were set to the normal value in a healthy population (91, 60, 100% and 100%, respectively). Descriptive statistics of patient demographic and clinical variables are shown in **Supplementary table 1**.

Muscle sample collection: Biopsy of the *vastus lateralis* muscle was performed under local anesthetic using a modified Bergstrom needle, as previously described [13]. Briefly, under sterile conditions, the skin and subcutaneous tissue overlying the lateral aspect of the distal third of the muscle were anesthetized, and a small incision was made in the outer fascial layer with a scalpel blade. The Bergstrom needle was advanced through the incision roughly 1 cm into the muscle, suction was applied as the trochar was advanced, and several pieces of muscle tissue were obtained. The needle was withdrawn under counter pressure, the skin closed with a single suture, and a pressure dressing applied. Tissue (approx. 200mg in total) was immediately sectioned with a sterile scalpel blade and pieces were processed for electron and light microscopy or flash frozen in liquid N2.

Muscle sample staining: Muscle biopsies were fixed in 10% buffered formalin phosphate for 24 hours at room temperature, rinsed in ethanol, paraffin embedded and sectioned (10 μm thickness) on cross section. The sections were rehydrated in a series of xylene and ethanol washes. Nuclei were stained with Weigert's haematoxylin for 8 minutes, washed in running tap water and then sections stained in picro-sirius red for one hour. Sections were then washed in two changes of acidified water, dehydrated with ethanol and xylene washes and mounted.

Gene set visualization using enrichment map: Enrichment map, a network-based visual representation of enriched terms that groups similar gene sets (functional terms, was performed to aid identification of functional themes (Supplementary Figure 2). Gene set functional enrichment analysis was performed using Gene Ontology (GO) biological process terms in the web-based version of gProfiler (http://biit.cs.ut.ee/gprofiler/). The statistical significance threshold level for GO enrichment was Benjamini and Hochberg corrected p < 0.05 and only gene sets between 10 and 100 genes were used from GO. The background list for the enrichment analysis included all genes represented on the Illumina Human HT-12 v4 array with a detection *P* value < 0.05 in at least three samples. Enrichment Map Cytoscape plugin software was used to create the enrichment map, with the parameters *p*-value < 0.005, correct p-value value < 0.05 and "Jaccard + overlap similarity" cutoff = 0.5.

Independent validation data sets*:* Two datasets were obtained from the National Center for Biotechnology Information (NCBI) Gene Expression Omnibus (GEO) database**.** *Validation dataset 1***:** Human skeletal muscle (*vastus lateralis*) transcriptome in ICU patients with sepsis induced multi-organ failure (GSE13205, 13 sepsis and 8 control samples) performed on Affymetrix GeneChip HG-U133 Plus 2.0 Array [14]. Expression data was age and sex corrected using residuals of a linear regression model. *Validation dataset 2:* Experimental model of ICUAW in *Sus scrofula* skeletal muscle (*biceps femoris*) transcriptome containing a total of 40 samples from a series of 5-day longitudinal experiments (24 samples, GSE16348; 8 samples GSE24239; 8 samples, GSE33037) performed

on Affymetrix Porcine Genome Array [15]. For both validation datasets, series raw data were downloaded (Affymetrix .CEL files) and background corrected using *rma* in the expresso package ("average difference" summary method) without normalization then arrays from the same platform were merged together and quantile normalized. Data quality was assessed as described above. Corresponding published putative homologs of the porcine dataset were used to link probe IDs to HUGO symbols [16].

References

1 Batt, J. *et al.* MEND ICU - Muscle Injury and Repair in Critical Illness Survivors Mechanically Ventilated for Over 7 Days. *Am J Respir Crit Med* **191**, A2288 (2015).

2 Ebert, S. M. *et al.* Stress-induced skeletal muscle Gadd45a expression reprograms myonuclei and causes muscle atrophy. *J. Biol. Chem.* **287**, 27290-27301, doi:10.1074/jbc.M112.374777 (2012).

3 Linacre, J. M., Heinemann, A. W., Wright, B. D., Granger, C. V. & Hamilton, B. B. The structure and stability of the Functional Independence Measure. *Arch. Phys. Med. Rehabil.* **75**, 127-132 (1994).

4 Kleyweg, R. P., van der Meche, F. G. & Schmitz, P. I. Interobserver agreement in the assessment of muscle strength and functional abilities in Guillain-Barre syndrome. *Muscle Nerve* **14**, 1103-1109, doi:10.1002/mus.880141111 (1991).

5 Marquis, K. *et al.* Midthigh muscle cross-sectional area is a better predictor of mortality than body mass index in patients with chronic obstructive pulmonary disease. *Am. J. Respir. Crit. Care Med.* **166**, 809-813, doi:10.1164/rccm.2107031 (2002).

6 Maughan, R. J., Watson, J. S. & Weir, J. Strength and cross-sectional area of human skeletal muscle. *J. Physiol* **338**, 37-49 (1983).

7 Goodpaster, B. H. *et al.* Attenuation of skeletal muscle and strength in the elderly: The Health ABC Study. *J. Appl. Physiol.* **90**, 2157-2165 (2001).

8 Kanehisa, H., Ikegawa, S. & Fukunaga, T. Comparison of muscle cross-sectional area and strength between untrained women and men. *Eur. J. Appl. Physiol. Occup. Physiol.* **68**, 148-154 (1994).

9 Segal, N. A., Zimmerman, M. B., Brubaker, M. & Torner, J. C. Obesity and knee osteoarthritis are not associated with impaired quadriceps specific strength in adults. *PM R* **3**, 314-323; quiz 323, doi:10.1016/j.pmrj.2010.12.011 (2011).

10 Hakkinen, K. *et al.* Bilateral and Unilateral Neuromuscular Function and Muscle Cross-Sectional Area in Middle-Aged and Elderly Men and Women. *The Journals of Gerontology Series A: Biological Sciences and Medical Sciences* **51A**, B21-B29, doi:10.1093/gerona/51A.1.B21 (1996).

11 Mathur, S., Makrides, L. & Hernandez, P. Test-retest reliability of isometric and isokinetic torque in patients with chronic obstructive pulmonary disease. *Physiother. Can.* **56**, 94-101 (2004).

12 Gosselink, R., Troosters, T. & Decramer, M. Peripheral muscle weakness contributes to exercise limitation in COPD. *Am. J. Respir. Crit. Care Med.* **153**, 976-980, doi:10.1164/ajrccm.153.3.8630582 (1996).

13 Bourgeois, J. M. & Tarnopolsky, M. A. Pathology of skeletal muscle in mitochondrial disorders. *Mitochondrion* **4**, 441-452, doi:10.1016/j.mito.2004.07.036 (2004).

14 Fredriksson, K. *et al.* Dysregulation of mitochondrial dynamics and the muscle transcriptome in ICU patients suffering from sepsis induced

multiple organ failure. *PLoS One* **3**, e3686, doi:10.1371/journal.pone.0003686 (2008).

15 Banduseela, V. C. *et al.* Impaired autophagy, chaperone expression, and protein synthesis in response to critical illness interventions in porcine skeletal muscle. *Physiol. Genomics* **45**, 477-486, doi:10.1152/physiolgenomics.00141.2012 (2013).

16 Tsai, S. *et al.* Annotation of the Affymetrix porcine genome microarray. *Anim. Genet.* **37**, 423-424, doi:10.1111/j.1365-2052.2006.01460.x (2006).

Module–trait relationships

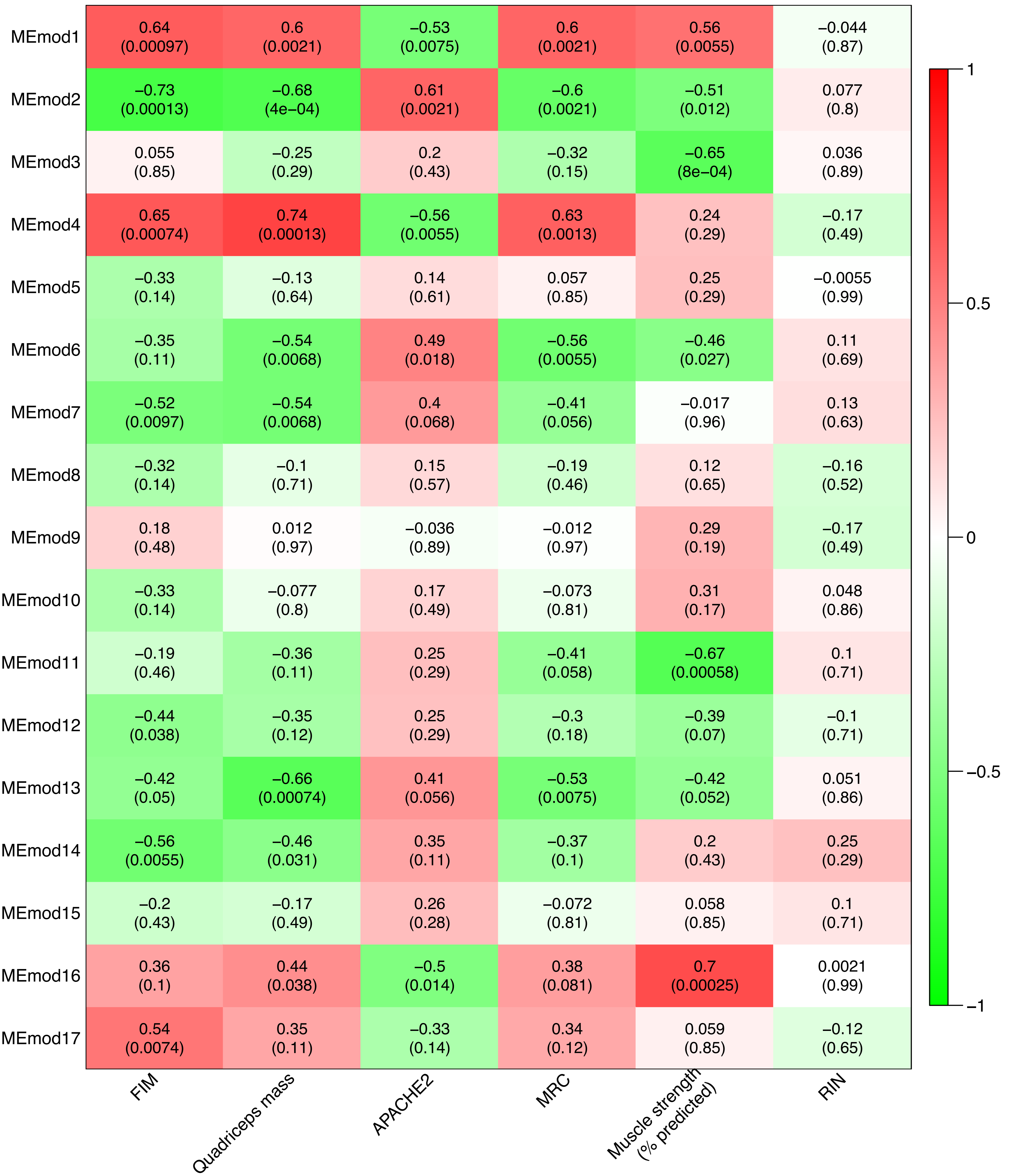

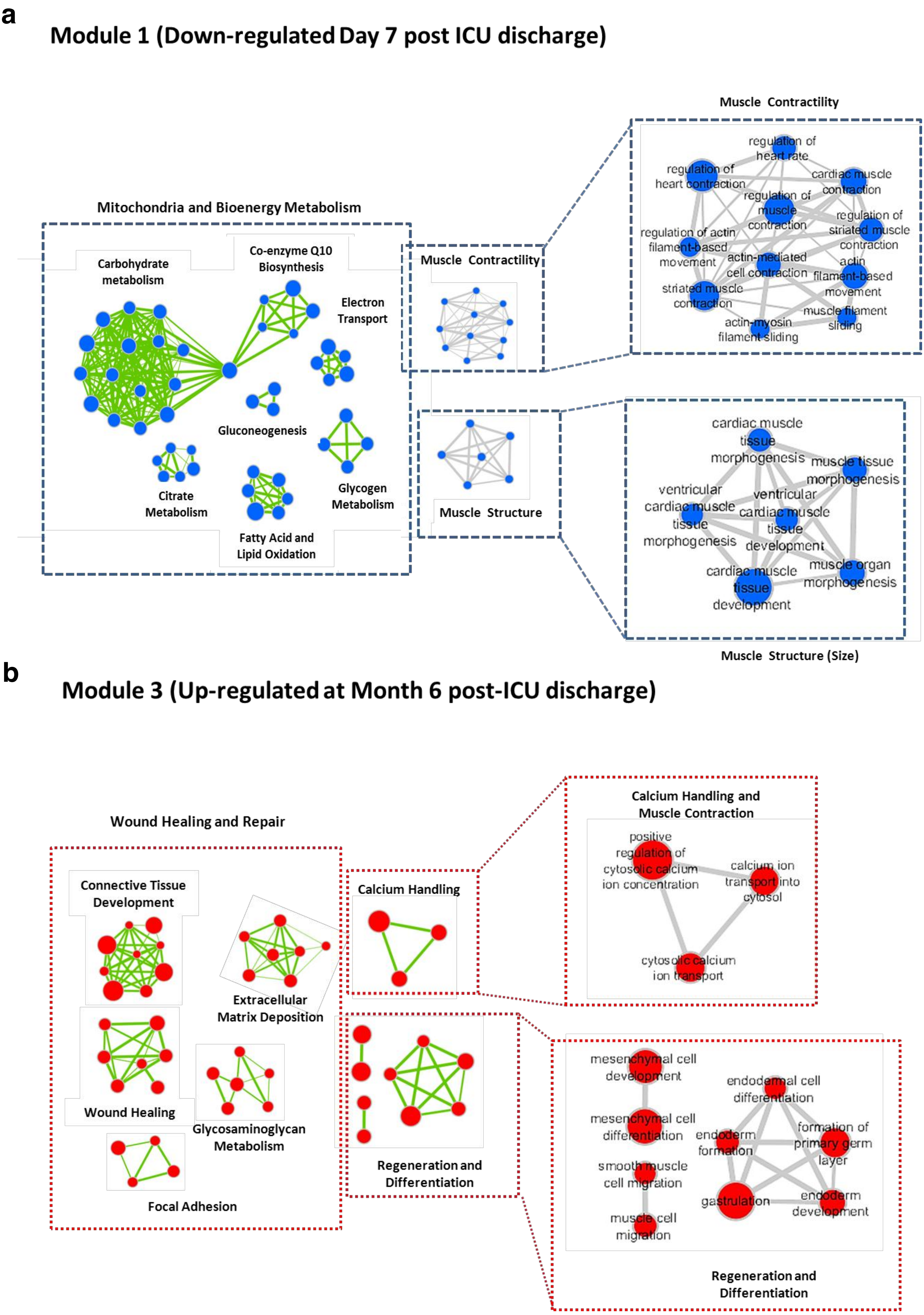

a
Module 1 (Down-regulated Day 7 post ICU discharge)
Mitochondria and Bioenergy Metabolism
Carbohydrate metabolism
Co-enzyme Q10 Biosynthesis
Electron Transport
Gluconeogenesis
Citrate Metabolism
Fatty Acid and Lipid Oxidation
Glycogen Metabolism
Muscle Contractility
Muscle Structure
Muscle Contractility
regulation of heart rate
regulation of heart contraction
cardiac muscle contraction
regulation of muscle contraction
regulation of striated muscle contraction
regulation of actin filament-based movement
actin-mediated cell contraction
actin filament-based movement
striated muscle contraction
muscle filament sliding
actin-myosin filament sliding
cardiac muscle tissue morphogenesis
muscle tissue morphogenesis
ventricular cardiac muscle tissue morphogenesis
ventricular cardiac muscle tissue development
cardiac muscle tissue development
muscle organ morphogenesis
Muscle Structure (Size)
b
Module 3 (Up-regulated at Month 6 post-ICU discharge)
Wound Healing and Repair
Connective Tissue Development
Extracellular Matrix Deposition
Wound Healing
Glycosaminoglycan Metabolism
Focal Adhesion
Calcium Handling
Regeneration and Differentiation
Calcium Handling and Muscle Contraction
positive regulation of cytosolic calcium ion concentration
calcium ion transport into cytosol
cytosolic calcium ion transport
mesenchymal cell development
endodermal cell differentiation
mesenchymal cell differentiation
endoderm formation
formation of primary germ layer
smooth muscle cell migration
gastrulation
endoderm development
muscle cell migration
Regeneration and Differentiation

Supplementary Table 1. Demographic and clinical differences for ICU acquired weakness patients (day 7 and month 6 post-ICU) and for healthy controls.

Data are presented as Median and Interquartile range (IQR) unless specified

	**Day 7 post ICU**	**Month 6 Post ICU**	**Control**	**P-value#**
Number of subjects	14	10	8	
Age (mean [standard deviation])	50.3 (15.9)	48.1 (13.5)	35.3 (9.5)	0.055
Sex	7F 7M	5F 5M	3F 5M	1
Quadriceps cross sectional area (percentage of age and sex matched norms)	40.2 (24.1)	58.2 (31.2)	100*	<0.001
Quadriceps strength (%predicted isometric peak torque)	NA	67.9 (18.6)	100*	<0.001
FIM motor subscore (max score 91)	32 (27.5)	83 (6.0)	91*	<0.001
MRCSS (max score 60)	50 (5.0)	56 (4.5)	60*	<0.001

* Clinical data values for controls are set to the normal value in a healthy population

Abbreviations: Medical Research Council Sum Score (MRCSS). Functional Independence Measure (FIM). Female (F), Male (M). Data not available (NA).

#Differences between groups for non-normally distributed variables were tested using Kruskal-Wallis one-way analysis of variance. Differences between groups for normally distributed variables were tested using one-way ANOVA. Differences in categorical data (sex) between groups were tested using Fisher's exact test.

Supplementary Table 2: Differentially expressed genes (N = 744 unique probes corresponding to 695 genes). LIMMA global analysis of differentially expressed genes between ICUAW and controls for both day 7 post-ICU and month 6 post-ICU at false discovery rate (FDR) 5% level was performed using data adjusted for age, sex, and correlation between patient samples.

Illumina ID	HGNC	Fold change ICUAW Day 7 vs. control	P value (FDR adjusted)	Fold change ICUAW Month 6 vs control	P value (FDR adjusted)
ILMN_1818859	HS.407666	-2.052	2.51E-06	-2.285	3.86E-07
ILMN_1710027	PNMT	1.368	0.00167	1.712	2.38E-06
ILMN_1883153	HS.543175	-1.707	1.27E-05	-1.748	1.11E-05
ILMN_1720745	LOC645385	1.715	1.15E-06	1.613	1.43E-05
ILMN_3245346	EFR3B	-1.874	6.30E-07	-1.703	1.68E-05
ILMN_1850374	HS.333785	-2.021	5.77E-05	-2.154	2.75E-05
ILMN_1903693	HS.573359	-1.684	6.55E-05	-1.77	2.95E-05
ILMN_1808825	OR51F1	-1.661	0.000339	-1.885	3.00E-05
ILMN_1897911	HS.565086	-2.101	5.01E-05	-2.182	4.02E-05
ILMN_1819158	HS.542632	-1.562	0.00353	-1.995	4.29E-05
ILMN_2283814	C21ORF34	-1.595	0.000169	-1.704	4.72E-05
ILMN_2157717	MYF6	-3.489	0.000362	-4.526	5.22E-05
ILMN_1893033	HS.544451	-1.936	7.12E-05	-2.01	5.35E-05
ILMN_1812461	WISP2	1.302	0.102	2.115	5.43E-05
ILMN_1854165	HS.564962	-1.425	0.000281	-1.516	5.66E-05
ILMN_1817048	HS.545044	-1.922	0.000337	-2.172	6.08E-05
ILMN_1710458	LOC646990	-2.083	0.000279	-2.34	6.80E-05
ILMN_1822180	HS.571435	-1.596	7.79E-05	-1.62	8.10E-05
ILMN_1803882	VEGFA	-2.745	1.51E-05	-2.517	8.52E-05
ILMN_1716583	NME7	1.783	3.97E-05	1.758	8.75E-05
ILMN_1718766	MT1F	1.624	0.000812	1.828	9.49E-05
ILMN_2405023	PPP1CB	-1.424	0.000483	-1.517	0.000102
ILMN_1750763	LOC643699	-1.662	0.000619	-1.844	0.000103
ILMN_1728202	TMEM22	1.348	0.00661	1.595	0.000109
ILMN_1769229	BCL2A1	-1.438	0.00159	-1.608	0.00012
ILMN_1728787	AGR3	-1.696	0.00027	-1.791	0.000124
ILMN_2073235	FTHL12	-2.093	1.08E-05	-1.889	0.000139
ILMN_1889144	HS.569953	-1.762	0.000689	-1.959	0.000143
ILMN_1757081	SYN2	-1.635	0.00142	-1.872	0.000144
ILMN_1651429	SELM	1.91	0.000408	2.064	0.00016
ILMN_1732318	PTK2B	-1.684	0.000323	-1.769	0.000163
ILMN_1874302	HS.543828	-1.897	0.000151	-1.927	0.00017
ILMN_1838702	HS.547858	-1.564	0.00303	-1.842	0.000174
ILMN_1904242	HS.114286	-1.641	0.000112	-1.636	0.000185
ILMN_1703408	FZD3	1.458	0.000516	1.528	0.000199
ILMN_1687842	PGA3	-1.965	0.000566	-2.138	0.000219
ILMN_1699836	LSP1	-1.515	0.000682	-1.605	0.000232
ILMN_2181892	BEX2	2.549	0.00173	3.217	0.000239
ILMN_1914284	HS.538083	-1.931	0.000341	-2.011	0.00025
ILMN_1706660	HYI	1.437	0.000501	1.489	0.000253
ILMN_1659306	SVIL	-1.378	0.00656	-1.597	0.000254
ILMN_1854029	HS.566801	-1.775	0.000419	-1.858	0.000256
ILMN_1824465	HS.148448	-2.032	8.32E-05	-1.952	0.000261
ILMN_1780057	RENBP	1.904	0.000131	1.873	0.000271
ILMN_1778505	LOC642771	-2.101	0.000581	-2.269	0.000284
ILMN_1859160	HS.117299	-1.785	0.000259	-1.811	0.000289
ILMN_1877455	HS.541892	-1.763	0.00203	-2.031	0.000289
ILMN_1832180	HS.557356	-1.711	0.000992	-1.861	0.000299
ILMN_1720373	SLC7A5	-1.642	0.00453	-1.972	0.000305
ILMN_2128428	DAB2	1.519	0.0137	1.946	0.000324
ILMN_2223941	FBLN5	1.354	0.00528	1.522	0.000328
ILMN_1777513	KCTD11	-1.345	0.00464	-1.497	0.000331
ILMN_3255124	ATL1	-1.627	0.000678	-1.709	0.000341
ILMN_1695058	SLC38A5	-1.83	0.000104	-1.762	0.000343

ILMN_1693270	SUSD2	1.362	0.0257	1.725	0.000343
ILMN_1874126	HS.544721	-1.41	0.0194	-1.78	0.000344
ILMN_1836172	HS.582136	-1.825	0.00216	-2.106	0.00035
ILMN_1832252	HS.369978	-1.631	0.000934	-1.738	0.000364
ILMN_1703233	LOC653382	-1.399	0.00358	-1.55	0.000371
ILMN_2132898	SPRN	-2.049	0.000181	-2.008	0.000379
ILMN_1706304	EIF2C4	-1.403	0.000618	-1.442	0.000383
ILMN_1705627	USP2	-1.734	0.000124	-1.678	0.00039
ILMN_1676361	ARHGAP22	1.568	0.000745	1.638	0.000394
ILMN_2358560	TIAM2	1.534	0.0157	1.975	0.000418
ILMN_1847029	HS.553290	-1.996	7.46E-05	-1.859	0.00042
ILMN_1668619	KIAA1467	1.314	0.00317	1.416	0.000421
ILMN_1772702	SFRS2B	1.177	0.0558	1.395	0.000425
ILMN_1807969	SNCAIP	1.295	0.0728	1.757	0.000438
ILMN_1877156	HS.567436	-1.795	0.00112	-1.939	0.000439
ILMN_1888925	HS.232535	-1.63	0.000212	-1.604	0.000459
ILMN_1879135	HS.555208	-1.694	0.00236	-1.894	0.000501
ILMN_3274671	LOC283481	1.754	1.70E-05	1.561	0.000519
ILMN_1657009	NFASC	-1.546	0.00068	-1.587	0.000524
ILMN_1772369	PDHA1	-1.613	0.000347	-1.608	0.00054
ILMN_1813581	CNR1	1.185	0.0951	1.478	0.000554
ILMN_3203534	LOC100132474	-1.413	0.00367	-1.547	0.000559
ILMN_1861915	HS.545663	-1.244	0.0316	-1.466	0.000565
ILMN_1763730	APPL1	-1.657	4.54E-06	-1.44	0.000569
ILMN_2396991	HCST	1.891	0.000189	1.818	0.000573
ILMN_1711422	PLEKHN1	-1.555	0.00274	-1.711	0.000574
ILMN_1740217	HACE1	-1.553	0.000136	-1.495	0.000575
ILMN_1699357	SLC22A5	-1.772	0.00578	-2.144	0.000578
ILMN_2303955	FKBP1B	-1.53	0.00121	-1.604	0.000581
ILMN_2375879	VEGFA	-2.678	2.74E-05	-2.219	0.00059
ILMN_1663422	RGL4	-1.419	0.000363	-1.413	0.000596
ILMN_1692785	KLHL21	-1.385	0.0154	-1.646	0.000616
ILMN_2365686	ALG8	1.789	6.23E-06	1.529	0.000619
ILMN_1907597	HS.553278	-1.614	0.00362	-1.818	0.000622
ILMN_1833415	LOC730877	-1.517	0.00016	-1.466	0.000633
ILMN_2134538	FTHL11	-2.085	4.69E-05	-1.846	0.000638
ILMN_1902998	HS.17661	-1.472	0.00125	-1.533	0.00064
ILMN_1713918	CYTH3	-1.632	0.000979	-1.696	0.000642
ILMN_2392674	PRR3	-1.539	0.000622	-1.56	0.000646
ILMN_1663417	C22ORF33	-1.575	0.00332	-1.748	0.000654
ILMN_1713496	ST3GAL5	-1.425	0.00755	-1.623	0.000655
ILMN_1880885	HS.545048	-2.103	0.000443	-2.097	0.000664
ILMN_1819590	HS.287720	-1.62	0.000539	-1.631	0.000669
ILMN_1738207	CISH	-2.057	0.00918	-2.731	0.000721
ILMN_1914579	HS.562118	-1.447	0.00159	-1.512	0.000731
ILMN_1690780	RFK	-1.339	0.00719	-1.48	0.000735
ILMN_3178792	HNRNPA2B1	-1.391	0.000297	-1.367	0.000764
ILMN_1796126	MXRA7	-1.367	0.0091	-1.541	0.000766
ILMN_1666690	ACRC	-1.664	0.000392	-1.64	0.00077
ILMN_1807181	BACH1	-1.544	0.00416	-1.716	0.000777
ILMN_1674243	TFRC	-5.05	8.49E-08	-2.485	0.000779
ILMN_1823270	HS.544326	-1.485	0.00369	-1.623	0.00078
ILMN_1685433	COL8A1	1.501	0.042	2.082	0.000785
ILMN_1839905	HS.544351	-1.277	0.00807	-1.392	0.000791
ILMN_1860789	HS.313056	-2.035	0.000218	-1.923	0.000799
ILMN_2340643	INSC	-1.999	7.00E-04	-2.029	0.000802
ILMN_1791728	SLC25A25	-2.632	0.00101	-2.778	0.000825
ILMN_1859259	HS.537451	-1.569	0.00127	-1.626	0.000838
ILMN_1651967	TP53I3	-1.645	0.0044	-1.855	0.000841
ILMN_1758895	CTSK	2.261	0.00088	2.334	0.000843
ILMN_3223843	LOC90499	-1.434	0.00242	-1.515	0.000866

ILMN_1796663	B4GALNT4	1.89	4.83E-05	1.676	0.000872
ILMN_1670037	POLR2L	1.351	0.0221	1.605	0.000876
ILMN_1803813	ASTE1	1.285	0.0409	1.56	0.000902
ILMN_1776213	RGMB	-1.444	0.0125	-1.689	0.000903
ILMN_1659270	OTP	-1.498	0.0034	-1.62	0.000914
ILMN_1903750	HS.544238	-1.751	0.000183	-1.651	0.000919
ILMN_1671123	LOC647543	1.711	0.0445	2.627	0.000928
ILMN_2408885	HDAC9	-1.661	0.000101	-1.541	0.000929
ILMN_2103720	MRPL15	-1.531	7.71E-05	-1.426	0.000951
ILMN_1768534	BHLHB2	-1.802	0.0195	-2.457	0.000953
ILMN_2229877	PCDH18	1.383	0.0322	1.721	0.000963
ILMN_1816948	HS.146561	-1.418	0.00459	-1.537	0.000976
ILMN_1689142	UBE1C	-1.487	0.000575	-1.476	0.000986
ILMN_1736093	SNX33	1.216	0.0326	1.388	0.000988
ILMN_1821883	HS.539119	-1.788	0.00173	-1.89	0.000993
ILMN_1795336	PTER	-2.519	8.45E-06	-1.928	0.001
ILMN_1755843	SLC26A8	-1.407	0.00499	-1.527	0.00101
ILMN_3244456	LOC100134122	-1.46	0.0019	-1.519	0.00102
ILMN_1765454	LOC732111	-1.325	0.00768	-1.446	0.00103
ILMN_1774589	IQCC	-1.644	0.000783	-1.648	0.00105
ILMN_1703475	LOC642981	-1.712	0.000628	-1.697	0.00105
ILMN_1770787	DDAH2	1.241	0.137	1.694	0.00105
ILMN_1743836	MXRA7	1.111	0.35	1.512	0.00105
ILMN_1653940	USP2	-1.451	0.00236	-1.522	0.00106
ILMN_1858507	HS.544228	-1.627	0.000919	-1.642	0.00107
ILMN_2400922	OPRL1	1.283	0.00412	1.349	0.00108
ILMN_1655296	UTRN	-1.939	0.0013	-2.009	0.00109
ILMN_1852279	HS.528210	-1.519	0.00102	-1.536	0.0011
ILMN_1719788	LDHAL6B	-1.574	0.00224	-1.657	0.00112
ILMN_2381603	ING3	1.179	0.0682	1.38	0.00113
ILMN_1850574	HS.545111	-1.518	0.00504	-1.668	0.00115
ILMN_1836060	HS.582211	-1.364	0.00575	-1.472	0.00116
ILMN_2220184	GFPT1	1.275	0.0162	1.422	0.00116
ILMN_1909197	HS.580452	1.273	0.0158	1.417	0.00116
ILMN_1785191	TMEM14A	1.431	0.0105	1.625	0.00116
ILMN_1790985	DJ341D10.1	-1.393	0.0148	-1.606	0.00117
ILMN_2078074	MUT	-1.447	0.00467	-1.564	0.00117
ILMN_1670708	F10	1.131	0.472	1.864	0.00117
ILMN_1861128	HS.543412	-1.489	0.0185	-1.802	0.00119
ILMN_1738707	S100A13	1.455	0.00899	1.641	0.00119
ILMN_1792384	HABP4	1.684	1.49E-05	1.458	0.0012
ILMN_3241607	LOC100132106	-1.81	0.000175	-1.671	0.00123
ILMN_1879739	HS.565146	-1.34	0.0131	-1.503	0.00123
ILMN_1672662	SLC20A1	-1.518	0.00543	-1.669	0.00123
ILMN_2071809	MGP	1.72	0.0213	2.269	0.00123
ILMN_1909895	HS.570330	-1.218	0.118	-1.563	0.00128
ILMN_3223966	LOC730254	-1.641	0.00265	-1.741	0.00128
ILMN_1719641	SMOC2	1.277	0.104	1.7	0.00129
ILMN_1745005	GGCT	-1.509	2.12E-05	-1.353	0.00132
ILMN_1851912	HS.385477	-1.402	0.00263	-1.457	0.00133
ILMN_2085922	WRB	-1.455	0.000831	-1.446	0.00137
ILMN_3261022	LOC100129906	-1.517	0.00455	-1.64	0.00138
ILMN_1882485	HS.554595	-1.458	6.89E-05	-1.349	0.0014
ILMN_1897230	HS.543617	-1.776	0.00346	-1.926	0.00143
ILMN_2088847	OTUD5	-1.25	0.0273	-1.414	0.00144
ILMN_1762106	MMP2	1.468	0.00192	1.506	0.00145
ILMN_3179396	LOC100129410	-1.493	0.00353	-1.581	0.00146
ILMN_1698334	LOC728863	-1.43	0.00156	-1.451	0.00147
ILMN_2344455	G3BP1	-1.242	0.0558	-1.48	0.00149
ILMN_1796431	GPR101	-1.807	0.00119	-1.817	0.0015
ILMN_1911873	HS.544751	-1.57	0.00217	-1.626	0.0015

ILMN_3237099	LOC100134815	-1.605	0.000314	-1.523	0.00152
ILMN_1857546	HS.575831	-1.416	0.00272	-1.467	0.00153
ILMN_2252136	YWHAE	-1.198	0.0629	-1.397	0.00156
ILMN_1702489	TRIM63	-1.879	0.0038	-2.056	0.00158
ILMN_1706410	HUS1B	-1.693	0.00254	-1.773	0.00162
ILMN_1795464	LTA	-1.451	0.000859	-1.434	0.00163
ILMN_1817816	HS.560319	-1.406	0.0219	-1.652	0.00164
ILMN_2171783	CPEB3	-1.281	0.0637	-1.579	0.00166
ILMN_1860753	HS.85445	-1.529	0.000378	-1.462	0.00168
ILMN_1838836	HS.282153	-1.557	0.00182	-1.587	0.00169
ILMN_1658743	CCNDBP1	1.65	4.16E-07	1.326	0.00172
ILMN_1685483	FETUB	-1.934	3.89E-05	-1.634	0.00173
ILMN_1822220	HS.128709	-1.379	0.00409	-1.443	0.00173
ILMN_1734010	C10ORF118	1.36	0.00577	1.443	0.00175
ILMN_2327947	SLC25A25	-1.874	0.00394	-2.041	0.00176
ILMN_1728349	TMEM63B	-1.274	0.0126	-1.382	0.00176
ILMN_1703079	NFS1	-1.247	0.0284	-1.403	0.00177
ILMN_1703511	PDZRN3	-1.507	0.0273	-1.867	0.00177
ILMN_2277523	DIP2A	-1.5	0.00341	-1.571	0.00178
ILMN_1672331	MAP3K7IP2	-1.3	0.0102	-1.402	0.0018
ILMN_2189842	SNORA10	-1.164	0.225	-1.54	0.0018
ILMN_1781819	PAPSS1	1.548	0.000211	1.445	0.00182
ILMN_1691090	MPV17	1.387	0.00427	1.453	0.00183
ILMN_1799600	STARD8	-1.387	0.00347	-1.438	0.00186
ILMN_1675721	UBE2R2	-1.24	0.0125	-1.329	0.00186
ILMN_1889215	HS.61208	-1.947	0.00263	-2.047	0.00187
ILMN_2306565	MTX2	-1.3	0.00404	-1.346	0.00187
ILMN_2401730	C1GALT1C1	1.689	1.96E-05	1.445	0.00188
ILMN_1774207	ANGPT2	2.043	0.000102	1.761	0.0019
ILMN_1678116	XAGE1E	-1.455	0.00129	-1.45	0.00192
ILMN_1773125	ENTPD1	-1.999	0.000952	-1.944	0.00195
ILMN_1690802	TRMT112	1.879	2.65E-06	1.475	0.00196
ILMN_3230241	LOC728975	-1.657	0.0018	-1.674	0.00204
ILMN_2197164	TAAR1	-1.507	0.000529	-1.44	0.00232
ILMN_3243034	LOC100134098	-1.384	0.00132	-1.372	0.00234
ILMN_1696911	FTHL8	-1.955	8.01E-05	-1.659	0.00245
ILMN_3201658	LOC642585	-1.595	0.000149	-1.447	0.00248
ILMN_2053415	LDLR	-2.176	1.25E-05	-1.67	0.00254
ILMN_1671905	C10ORF78	1.547	0.00161	1.535	0.00256
ILMN_1748034	KLHDC4	1.623	0.000153	1.463	0.00269
ILMN_2174574	HNRNPA3P1	-1.956	2.42E-06	-1.484	0.00282
ILMN_1916513	HS.572883	-1.569	0.00166	-1.549	0.0029
ILMN_1781626	C1S	1.697	0.000885	1.618	0.0029
ILMN_2407389	GPNMB	2.405	8.92E-06	1.744	0.00297
ILMN_1859701	HS.545514	-1.634	0.000483	-1.522	0.00301
ILMN_1732410	SLC16A9	2.371	0.000155	1.954	0.00304
ILMN_1694466	ZBED1	-1.468	0.00166	-1.449	0.00305
ILMN_3292114	LOC100131510	-1.682	9.07E-05	-1.471	0.00309
ILMN_1727177	MGAM	-2.132	2.29E-05	-1.662	0.0031
ILMN_1701386	STRADB	-1.845	1.31E-06	-1.409	0.00315
ILMN_2071446	PI15	-1.427	8.57E-05	-1.299	0.00323
ILMN_1766094	MOSPD2	1.464	0.000156	1.34	0.0033
ILMN_1658460	LOC653884	1.561	8.93E-05	1.387	0.00332
ILMN_3230880	BEND7	-1.644	0.000101	-1.445	0.00337
ILMN_1804833	LOC645039	-1.474	0.000298	-1.367	0.00346
ILMN_1776493	MTUS1	-1.428	0.00121	-1.386	0.00361
ILMN_3234124	C17ORF101	1.463	0.00104	1.408	0.00368
ILMN_1715543	ACOT1	-1.584	0.000202	-1.426	0.0038
ILMN_1746948	MYL5	4.574	0.00113	3.943	0.00385
ILMN_1716237	ACOT2	-1.736	2.78E-05	-1.436	0.00416
ILMN_2107991	HABP4	1.803	6.45E-05	1.509	0.00416

ILMN_2345016	PTGES2	-1.418	0.000748	-1.349	0.0042
ILMN_1681754	GGH	1.731	0.000878	1.608	0.00437
ILMN_1667577	LCMT2	1.44	0.000231	1.323	0.00438
ILMN_1904843	HS.129547	-1.47	0.000709	-1.386	0.00442
ILMN_2173835	FTHL3	-1.795	0.000499	-1.61	0.00465
ILMN_1785891	PRKD1	1.615	0.000934	1.514	0.00466
ILMN_1654016	MRLC2	1.519	2.55E-05	1.308	0.00467
ILMN_2203588	MYL5	4.935	0.00126	4.127	0.00476
ILMN_1757287	MAPK6	-1.557	0.000381	-1.42	0.00479
ILMN_1750008	SUPV3L1	-1.429	0.000225	-1.309	0.00487
ILMN_1733535	ZNF366	-1.929	5.45E-05	-1.558	0.00492
ILMN_1790741	RNF126	-1.525	0.000137	-1.357	0.00493
ILMN_1658437	SFXN4	-1.397	0.000307	-1.293	0.00519
ILMN_1870085	HS.128892	-1.688	0.00125	-1.582	0.00524
ILMN_1699772	RRAGD	-1.423	0.0013	-1.363	0.00533
ILMN_3268813	LOC100130934	-1.626	0.00161	-1.547	0.00534
ILMN_3250067	ANGPT2	2.114	0.000572	1.834	0.00534
ILMN_1704793	MYPOP	-1.616	4.79E-05	-1.371	0.00557
ILMN_2409167	ANXA2	1.634	0.00187	1.558	0.0058
ILMN_1884357	HS.137293	-1.692	0.00151	-1.59	0.00582
ILMN_1687351	ANKRA2	1.768	4.51E-06	1.374	0.00586
ILMN_2250445	SMTN	-1.662	3.09E-05	-1.378	0.006
ILMN_1723978	LGALS1	1.491	0.000272	1.346	0.0062
ILMN_1742269	LOC642726	-1.681	0.00117	-1.552	0.00644
ILMN_2401258	FAM13A	1.721	0.000162	1.467	0.00665
ILMN_1849997	HS.436627	-1.681	0.0015	-1.567	0.00671
ILMN_1727618	C8ORF38	-1.434	0.000677	-1.331	0.007
ILMN_2358919	TP53I3	2.14	0.000189	1.713	0.00714
ILMN_1775008	NCAPD2	1.586	0.00167	1.49	0.00725
ILMN_3293244	LOC100131744	-1.353	0.00153	-1.295	0.00728
ILMN_1757660	CAPS	-1.374	0.00102	-1.298	0.00733
ILMN_1672554	C17ORF81	-1.46	9.42E-05	-1.285	0.00766
ILMN_1778951	C6ORF203	-1.47	3.00E-04	-1.324	0.00768
ILMN_1890788	HS.534997	-1.522	0.00177	-1.437	0.0078
ILMN_1702247	CCNDBP1	1.64	5.75E-06	1.309	0.0078
ILMN_1651643	ASB11	-1.654	0.000224	-1.428	0.00782
ILMN_2215640	TUBA3D	-2.235	0.000316	-1.794	0.00801
ILMN_1805636	PGAP3	1.491	0.00106	1.383	0.00812
ILMN_1718961	BNIP3L	1.395	0.00089	1.303	0.00826
ILMN_2403237	CHN2	1.905	0.000264	1.582	0.0083
ILMN_1774287	CFB	1.707	0.00033	1.474	0.00831
ILMN_1752510	FAM13A	1.611	0.000418	1.424	0.00833
ILMN_1892608	HS.76704	-1.482	0.00182	-1.397	0.00887
ILMN_1808792	ALKBH6	1.438	0.000978	1.334	0.00906
ILMN_2367275	BCL7B	-1.401	0.000524	-1.286	0.0091
ILMN_3247023	FLJ22536	1.716	0.000583	1.503	0.0091
ILMN_1735822	TTC30A	1.651	7.14E-05	1.372	0.00925
ILMN_1806601	GRSF1	-1.437	9.70E-05	-1.263	0.00931
ILMN_1782167	RPL32	1.448	8.86E-05	1.267	0.00941
ILMN_1801348	GOT2	-2.048	4.84E-06	-1.455	0.00955
ILMN_1679150	LOC387647	-1.857	0.00093	-1.621	0.00981
ILMN_3233388	RELL1	-1.512	0.000357	-1.341	0.0102
ILMN_1718520	C10ORF59	-1.781	8.10E-05	-1.436	0.0104
ILMN_1703335	LACTB	-1.488	0.00044	-1.331	0.0105
ILMN_1814924	FAM55C	-1.691	0.000261	-1.433	0.0107
ILMN_1828245	HS.542149	-1.485	0.000946	-1.357	0.0107
ILMN_2336647	NNT	-1.802	2.97E-05	-1.407	0.0107
ILMN_1712754	NFKBIB	-1.441	0.000955	-1.322	0.0114
ILMN_2104877	CMPK1	-1.342	0.0014	-1.264	0.0115
ILMN_1811754	NDUFB10	-1.566	6.51E-06	-1.262	0.0115
ILMN_2153485	NMNAT3	-1.737	9.45E-05	-1.412	0.0115

ILMN_2336133	SULT1A4	1.423	0.000623	1.295	0.0115
ILMN_1754244	MYH8	12.6	0.000134	5.056	0.0117
ILMN_1712530	AKAP1	-1.612	0.000649	-1.419	0.0118
ILMN_1807359	CLEC11A	1.568	0.00191	1.444	0.0118
ILMN_1741180	HEXDC	-1.455	0.00107	-1.334	0.0119
ILMN_1704369	LIMA1	1.576	0.00115	1.422	0.0119
ILMN_1690085	STK11IP	1.389	0.000533	1.266	0.0119
ILMN_1752199	LHPP	1.377	0.000601	1.261	0.0122
ILMN_3247835	CXORF64	1.695	0.00122	1.505	0.0124
ILMN_1763941	LRRC49	1.413	0.000546	1.279	0.0126
ILMN_1745152	UQCC	-1.434	0.00059	-1.295	0.0127
ILMN_3182171	FGGY	1.931	0.000504	1.593	0.0127
ILMN_2307656	AGTRAP	2.38	2.86E-05	1.629	0.0128
ILMN_2368773	FAM3C	1.443	0.000578	1.3	0.0128
ILMN_1796244	CD2BP2	1.682	2.01E-06	1.278	0.0132
ILMN_1764043	TTL	-1.566	0.0016	-1.424	0.0133
ILMN_1718853	UQCRC2	-1.381	0.000522	-1.255	0.0135
ILMN_1790891	CKAP4	1.424	0.00139	1.313	0.014
ILMN_1729130	C7ORF42	1.547	0.000196	1.323	0.0141
ILMN_1739496	PRRX1	1.552	0.00191	1.415	0.0148
ILMN_2183784	TTC12	1.593	0.000782	1.397	0.015
ILMN_1745779	TCTEX1D2	1.532	0.000279	1.321	0.0151
ILMN_1748077	DDX59	-1.57	0.000465	-1.36	0.0152
ILMN_1795839	SCCPDH	-1.522	0.00146	-1.378	0.0152
ILMN_1801205	GPNMB	2.524	2.09E-05	1.642	0.0152
ILMN_2326512	CASP1	1.685	0.000186	1.389	0.0153
ILMN_2305225	NDRG4	-1.761	9.03E-05	-1.399	0.0155
ILMN_1796773	BTBD8	-2.057	4.10E-05	-1.496	0.0156
ILMN_1802089	SYMPK	1.547	0.000453	1.344	0.0157
ILMN_1724266	LYPD2	-1.422	0.000643	-1.279	0.0159
ILMN_1847612	HS.543981	-1.407	0.00115	-1.287	0.016
ILMN_3205656	LOC391075	-1.519	0.000654	-1.339	0.0161
ILMN_1742789	LPXN	1.545	0.000232	1.318	0.0162
ILMN_1708516	PRTFDC1	1.408	0.00136	1.293	0.0162
ILMN_1858599	HS.20255	1.487	0.0012	1.341	0.0163
ILMN_1767816	APH1B	1.451	0.000728	1.299	0.0165
ILMN_1704079	RBM38	-1.588	0.00152	-1.418	0.0166
ILMN_2121282	MRPS18B	-1.618	1.01E-05	-1.272	0.0171
ILMN_3200421	LOC641746	-1.78	0.000114	-1.408	0.0174
ILMN_1732187	TMEM143	-1.899	0.000146	-1.473	0.0174
ILMN_1723834	FLJ32011	-1.456	0.00155	-1.326	0.0176
ILMN_1683133	KLF15	-1.715	0.000622	-1.447	0.0176
ILMN_3265228	LOC100128392	-1.703	0.000754	-1.448	0.0177
ILMN_1652379	SUCLG2	-1.468	0.000359	-1.283	0.0179
ILMN_2173611	MT1E	2.994	0.000329	2.024	0.0181
ILMN_1673535	LOC340156	-3.739	4.68E-05	-2.065	0.0182
ILMN_1805826	BIVM	-1.568	4.13E-05	-1.277	0.0184
ILMN_1766054	ABCA1	2.406	1.50E-06	1.472	0.0184
ILMN_1677452	REXO4	-1.514	0.00188	-1.37	0.0185
ILMN_1745041	TCF15	-1.649	0.00011	-1.34	0.0185
ILMN_3248069	LOC653881	2.068	0.000121	1.532	0.0188
ILMN_1733998	DHRS9	3.387	2.21E-05	1.881	0.0189
ILMN_1745034	SLC11A2	-1.7	0.000111	-1.361	0.0192
ILMN_3236259	PPIAL4A	1.762	0.000226	1.417	0.0194
ILMN_1703370	ZDHHC12	1.554	9.72E-05	1.288	0.0194
ILMN_1729142	CENPV	-1.81	0.000614	-1.489	0.0197
ILMN_1724533	LY96	2.094	0.000848	1.667	0.0197
ILMN_1682993	NKG7	2.604	0.000161	1.765	0.0202
ILMN_1800103	LOC731196	-1.547	0.000273	-1.309	0.0207
ILMN_1729509	C1ORF43	-1.41	0.000164	-1.225	0.021
ILMN_2397880	CSTF3	1.422	0.00151	1.291	0.021

ILMN_1775182	GSR	-1.5	0.000832	-1.317	0.0215
ILMN_1663605	RNF123	-1.324	0.00128	-1.22	0.0219
ILMN_1704477	COX5A	-1.387	0.000172	-1.212	0.0223
ILMN_1838863	HS.497591	-1.602	0.000941	-1.38	0.0223
ILMN_1761309	ADCK5	1.488	0.000221	1.269	0.0224
ILMN_2048607	ANKRD9	-1.414	0.000979	-1.268	0.0226
ILMN_3244650	FLJ41941	-3.582	0.000157	-2.098	0.0227
ILMN_1691611	LOC645436	1.48	0.000693	1.295	0.023
ILMN_1725899	HOXC10	-1.514	0.00105	-1.329	0.0232
ILMN_1667670	SLC25A15	-1.546	0.00072	-1.334	0.0233
ILMN_1667418	LOC283953	-1.712	0.000868	-1.434	0.0235
ILMN_1813938	CHCHD4	-1.405	0.000251	-1.226	0.0237
ILMN_3264066	LOC100129720	-1.403	0.00154	-1.272	0.0239
ILMN_1709635	SLC38A11	-1.527	0.00101	-1.333	0.0242
ILMN_1777644	PIB5PA	-2.344	6.16E-06	-1.475	0.0243
ILMN_1815882	HNRNPA1	1.512	0.000606	1.305	0.0244
ILMN_1707475	UBE2E2	1.458	0.000289	1.255	0.0245
ILMN_1805225	LPCAT3	1.688	0.000102	1.334	0.0252
ILMN_2128967	C11ORF1	1.851	8.60E-05	1.396	0.0254
ILMN_2404049	RBM38	-1.522	0.0019	-1.352	0.0256
ILMN_1749403	TSPAN33	-1.484	0.001	-1.303	0.0256
ILMN_1791388	ZNF787	-1.457	0.00067	-1.273	0.0264
ILMN_2367428	FAM96A	-1.825	0.000281	-1.428	0.0266
ILMN_1731233	GZMH	2.274	0.00053	1.671	0.027
ILMN_1739886	HNF4A	-2.04	0.00012	-1.48	0.0271
ILMN_1788099	LSM4	1.372	0.00171	1.247	0.0276
ILMN_1741475	C7ORF47	1.576	0.000164	1.291	0.0277
ILMN_2156172	HK2	-1.892	0.000458	-1.477	0.028
ILMN_1670901	COX10	-1.831	3.41E-05	-1.35	0.0283
ILMN_2360291	UGCGL1	1.46	0.000571	1.266	0.0283
ILMN_1704398	FZD9	-1.673	0.0013	-1.414	0.0287
ILMN_1701998	AFAP1	1.673	0.00186	1.434	0.0288
ILMN_3239568	MYLK4	-3.616	0.000123	-2.012	0.0289
ILMN_1708496	BRSK2	-2.232	0.000379	-1.614	0.0293
ILMN_2334296	IL18BP	1.75	0.000322	1.388	0.0296
ILMN_1714861	CD68	2.134	0.00178	1.686	0.0303
ILMN_1710756	ENO1	1.465	0.000271	1.245	0.0305
ILMN_2075334	HIST1H4C	1.419	0.000857	1.25	0.0306
ILMN_1851107	HS.280924	-1.575	0.000123	-1.277	0.0307
ILMN_1740590	LOC647881	-1.739	0.000462	-1.394	0.031
ILMN_2347068	MKNK2	-1.638	0.00141	-1.391	0.031
ILMN_3291053	LOC346085	-1.655	0.000677	-1.368	0.0311
ILMN_2097858	KIAA1737	-1.614	0.00128	-1.372	0.0313
ILMN_2094166	CHMP5	1.57	0.000126	1.274	0.0314
ILMN_1660602	C1ORF43	-1.411	0.000597	-1.235	0.0315
ILMN_2050255	UCKL1	-1.398	0.000516	-1.224	0.0318
ILMN_1749409	LOC649864	-2.184	0.000286	-1.563	0.032
ILMN_3182120	LOC100129522	1.672	0.000881	1.385	0.0321
ILMN_1683023	PDGFC	1.823	0.000288	1.409	0.0323
ILMN_2319994	RPL3	1.841	0.00113	1.485	0.0325
ILMN_1792110	C10ORF76	-1.906	1.21E-05	-1.335	0.0331
ILMN_1782635	YARS2	-1.37	0.00122	-1.226	0.0341
ILMN_1667977	TAF1B	1.49	0.000287	1.252	0.0341
ILMN_2225144	EIF4E3	-1.553	0.000302	-1.283	0.0346
ILMN_1669669	KCMF1	-1.442	0.000924	-1.258	0.0347
ILMN_1778478	CCDC28B	-1.758	0.00077	-1.415	0.0348
ILMN_2180315	ATG4D	-1.381	0.000655	-1.216	0.0349
ILMN_1751206	MLLT10	-2.194	0.000208	-1.536	0.035
ILMN_1759396	NNT	-1.822	3.45E-05	-1.33	0.0353
ILMN_1731699	RAB15	1.998	0.00109	1.549	0.0358
ILMN_1902571	HS.557622	-1.696	0.000169	-1.323	0.0374

ILMN_1722309	ENDOG	-1.829	3.53E-05	-1.327	0.038
ILMN_1810392	ZNHIT2	1.547	0.000225	1.266	0.0384
ILMN_1736940	HPRT1	1.668	4.07E-05	1.273	0.039
ILMN_1767129	ABCC8	1.796	0.00028	1.376	0.0399
ILMN_1675421	LOC389293	-1.735	0.00115	-1.409	0.04
ILMN_1666553	SLC25A19	-1.483	0.000969	-1.272	0.04
ILMN_2385647	ALAS1	-2.162	1.47E-06	-1.338	0.0404
ILMN_1764410	C22ORF13	-1.531	0.000189	-1.251	0.0406
ILMN_2322842	PPHLN1	1.531	0.000714	1.286	0.0407
ILMN_1703005	IFP38	-1.413	0.00127	-1.241	0.0408
ILMN_1683576	MAGED2	1.669	0.00101	1.367	0.0408
ILMN_1787344	LOC652078	-1.374	0.00134	-1.221	0.0411
ILMN_3307930	RAN	1.379	0.000327	1.192	0.0416
ILMN_3281599	LOC642741	1.831	0.00113	1.45	0.0421
ILMN_3250389	LOC440895	1.628	2.22E-05	1.239	0.044
ILMN_1718852	PLCL1	-1.616	0.0016	-1.353	0.0443
ILMN_1748916	C18ORF55	-1.625	0.000122	-1.273	0.0445
ILMN_1698307	DBNL	1.578	0.000295	1.276	0.0445
ILMN_1786612	PSME2	1.573	0.000101	1.248	0.0447
ILMN_1798485	ATP6V1E1	1.438	0.00104	1.242	0.0459
ILMN_2345319	PREPL	1.694	0.000369	1.33	0.0459
ILMN_2244841	ALDH4A1	1.836	0.000539	1.405	0.046
ILMN_1779616	SUCLG1	-1.54	7.09E-05	-1.226	0.0465
ILMN_1787109	CLK2	1.397	0.00116	1.222	0.0473
ILMN_1764266	CKMT2	-1.948	0.00011	-1.383	0.0476
ILMN_1666306	SRRD	-1.534	0.00032	-1.255	0.0476
ILMN_1673026	CHCHD3	-1.6	0.000167	-1.266	0.0477
ILMN_2376502	RHOBTB1	-1.668	0.0019	-1.38	0.0479
ILMN_1652357	PDHX	-1.454	0.00133	-1.254	0.049
ILMN_1759023	WFS1	1.422	0.00131	1.236	0.0496
ILMN_1700349	ADCK4	1.513	0.000316	1.243	0.0497
ILMN_2085844	GXYLT2	-2.086	4.73E-05	-1.393	0.05
ILMN_1812777	MRPL35	-1.423	0.000583	-1.215	0.0503
ILMN_1772261	GLG1	1.587	0.000141	1.254	0.0503
ILMN_1722622	CD163	1.968	0.00191	1.524	0.0507
ILMN_3247821	TMEM206	1.44	0.000372	1.213	0.0507
ILMN_1801996	MASP1	-3.231	1.94E-06	-1.533	0.0512
ILMN_2408001	RFWD2	-1.314	0.00104	-1.173	0.0512
ILMN_2207328	C18ORF10	1.802	7.38E-05	1.313	0.0518
ILMN_1714536	SCGB1D2	-3.46	1.28E-05	-1.652	0.0542
ILMN_1695717	RBM41	-1.412	0.000855	-1.215	0.0544
ILMN_1664921	PPP6C	-1.377	0.000404	-1.183	0.0551
ILMN_1758827	RTN4IP1	-1.63	0.000295	-1.282	0.0557
ILMN_1692896	JMJD4	1.476	0.000878	1.243	0.0564
ILMN_1745271	EXOSC4	1.628	0.00152	1.334	0.0568
ILMN_1726114	SLC45A3	-2.267	0.000839	-1.577	0.0569
ILMN_2109708	ECGF1	1.746	0.000583	1.349	0.0569
ILMN_1733690	AKAP7	-1.985	0.000661	-1.451	0.0572
ILMN_1715508	NNMT	6.627	0.00039	2.657	0.0574
ILMN_1665123	NMNAT3	-1.57	0.00166	-1.308	0.0575
ILMN_2138801	TP73L	1.94	0.000216	1.384	0.0575
ILMN_2342695	PDGFA	-1.591	0.000809	-1.293	0.0577
ILMN_1786278	FAM149A	-2.402	0.000124	-1.506	0.0583
ILMN_1661804	CTF1	-1.509	0.00049	-1.241	0.0587
ILMN_1663532	RIC8B	-1.601	7.24E-05	-1.234	0.0589
ILMN_2355665	MTP18	-2.38	0.00159	-1.664	0.0594
ILMN_1652006	SMC1A	1.405	0.000493	1.195	0.0594
ILMN_1774272	ESRRA	-1.62	0.000154	-1.256	0.0598
ILMN_1652906	GBGT1	1.324	0.00145	1.176	0.0611
ILMN_1740633	PRF1	1.556	0.000997	1.276	0.0627
ILMN_1700081	FST	5.63	0.000403	2.401	0.0628

ILMN_2406892	C19ORF2	-1.463	0.00015	-1.194	0.0631
ILMN_2240009	ADSSL1	-2.054	0.00153	-1.512	0.0641
ILMN_1728049	S100A16	1.657	0.000997	1.319	0.0647
ILMN_1741599	MEMO1	-1.448	0.000466	-1.207	0.0648
ILMN_1689828	DMPK	2.21	0.000285	1.47	0.0658
ILMN_1656186	SLC41A1	-1.944	0.000185	-1.365	0.066
ILMN_1693310	ITFG1	1.489	3.96E-05	1.18	0.066
ILMN_2345739	CAPRIN2	1.99	1.16E-05	1.3	0.0666
ILMN_1712067	CCDC135	-1.413	0.00168	-1.219	0.067
ILMN_1741148	ALDOA	-1.613	7.43E-05	-1.23	0.0673
ILMN_1751753	IDH2	-1.647	0.000232	-1.267	0.0677
ILMN_3253126	FLJ41484	3.161	0.000127	1.676	0.0693
ILMN_1752728	FUCA1	1.75	0.000267	1.305	0.0695
ILMN_1738866	DEXI	-1.558	0.000231	-1.23	0.0716
ILMN_3283772	LOC644237	-1.499	0.00189	-1.26	0.0717
ILMN_1705397	PDK2	-1.851	0.000481	-1.356	0.0734
ILMN_1669409	VSIG4	1.619	0.00189	1.314	0.0734
ILMN_2393341	LIAS	-1.632	0.00017	-1.247	0.074
ILMN_1765746	SFT2D3	1.476	0.00117	1.233	0.0741
ILMN_1813344	C20ORF7	-1.507	0.00102	-1.242	0.0751
ILMN_1810228	TTF2	2.499	5.86E-05	1.459	0.076
ILMN_2099586	CCDC28B	-1.717	0.00162	-1.347	0.0765
ILMN_3213792	LOC439953	1.614	0.000745	1.276	0.0771
ILMN_3246608	CENPV	-1.745	0.00143	-1.352	0.0781
ILMN_1703324	PDSS1	-1.62	0.000177	-1.24	0.0781
ILMN_1681503	MCM2	1.689	0.000179	1.263	0.0784
ILMN_1761084	FNDC5	-2.019	0.00103	-1.443	0.0785
ILMN_1810875	SYNGR1	-1.684	0.000264	-1.271	0.0785
ILMN_1655694	LOC642031	-1.58	0.000173	-1.224	0.0801
ILMN_1801045	SPIN3	1.349	0.00125	1.172	0.0805
ILMN_2350607	C20ORF7	-1.555	0.00168	-1.272	0.0807
ILMN_1672081	NEURL	-1.718	0.00175	-1.342	0.0827
ILMN_1814823	FTL	1.637	0.000998	1.287	0.0837
ILMN_3193306	C14ORF109	-1.446	0.000419	-1.189	0.0846
ILMN_2409055	FAM13C1	2.061	0.000791	1.432	0.0856
ILMN_1772964	CCL8	1.881	0.000796	1.367	0.0869
ILMN_1794230	SCAND1	1.442	0.00175	1.217	0.0872
ILMN_1779324	GZMA	2.003	0.00113	1.427	0.0875
ILMN_2052331	MYOC	-2.382	0.000656	-1.523	0.0876
ILMN_2219556	ISCA1	-1.485	0.000971	-1.217	0.0911
ILMN_1785892	MASP1	-3.601	6.55E-07	-1.456	0.0928
ILMN_1731851	OXA1L	-1.445	0.000205	-1.171	0.0933
ILMN_1806349	SLC6A8	-1.76	1.65E-05	-1.222	0.0951
ILMN_3237286	LOC100132794	-2.261	5.73E-05	-1.369	0.0957
ILMN_1713985	MAF1	-1.347	0.000603	-1.149	0.0974
ILMN_2405305	ARNTL	2.342	0.000809	1.503	0.0977
ILMN_1662640	C20ORF127	2.355	0.000735	1.501	0.0979
ILMN_3271630	FGGY	1.772	0.000284	1.282	0.0987
ILMN_1653466	HES4	-1.891	0.000797	-1.354	0.0991
ILMN_1747759	WSB1	1.433	0.00125	1.195	0.101
ILMN_1712430	ATP5G1	-2.616	0.00189	-1.643	0.102
ILMN_1745368	TMEM50A	1.501	0.000934	1.215	0.102
ILMN_1667079	SPTBN2	-2.028	0.000155	-1.334	0.103
ILMN_1804328	WWP1	-1.542	0.000456	-1.213	0.104
ILMN_2080637	ZBTB44	-1.497	0.00164	-1.226	0.104
ILMN_1787526	MGC13057	-1.916	0.000397	-1.33	0.105
ILMN_2355042	CLUAP1	1.342	0.000919	1.15	0.105
ILMN_3225941	LOC728368	1.527	0.00132	1.231	0.105
ILMN_1755462	UGCGL1	1.562	7.20E-05	1.185	0.106
ILMN_1735495	TBC1D8	-1.717	0.00144	-1.305	0.108
ILMN_1779040	INO80B	1.651	0.000612	1.254	0.108

ILMN_1783681	MRPL34	-1.509	0.0017	-1.226	0.111
ILMN_2329927	ABCG1	2.271	0.00074	1.45	0.113
ILMN_2412927	GMPPB	1.569	0.000731	1.226	0.113
ILMN_1801504	RUNX1	3.376	0.000189	1.626	0.115
ILMN_1761981	FAM96A	-1.763	5.11E-05	-1.225	0.117
ILMN_1777129	C16ORF56	1.393	0.000767	1.161	0.117
ILMN_1771957	MAN1B1	1.412	0.00161	1.182	0.117
ILMN_1903345	HS.575085	-1.375	0.000641	-1.151	0.119
ILMN_2049021	PTTG3P	1.933	0.000826	1.346	0.119
ILMN_2073289	MTSS1	1.81	7.75E-05	1.244	0.12
ILMN_1700047	ALAS1	-1.793	0.000204	-1.259	0.121
ILMN_1798712	USP4	-1.337	0.000795	-1.139	0.121
ILMN_1654690	CECR5	-1.564	0.000236	-1.195	0.122
ILMN_1721669	IDH3B	-1.848	3.60E-05	-1.236	0.122
ILMN_1737604	FLJ10986	1.964	0.000962	1.358	0.122
ILMN_1719468	EPM2A	-1.382	0.00163	-1.167	0.123
ILMN_2377669	CD247	1.851	0.000993	1.322	0.123
ILMN_1769520	UBE2L6	1.624	0.00021	1.208	0.127
ILMN_2261416	CD3D	1.652	0.00148	1.263	0.129
ILMN_1803856	DKFZP586I1420	1.669	0.000821	1.251	0.129
ILMN_1790549	TSPAN3	1.509	0.000475	1.187	0.129
ILMN_1802458	AGTRAP	1.856	0.000698	1.305	0.13
ILMN_1698934	CMTM7	2.819	0.000392	1.525	0.13
ILMN_1801553	LEO1	1.487	0.00131	1.199	0.131
ILMN_2412564	NCBP2	1.608	0.000218	1.201	0.131
ILMN_1716264	ANKRD1	2.477	0.000674	1.472	0.132
ILMN_3235065	ZNHIT6	1.321	0.00152	1.137	0.132
ILMN_1656951	APCDD1	-1.676	0.00116	-1.26	0.133
ILMN_1799488	ZNF383	-1.459	0.000758	-1.176	0.133
ILMN_2175114	KCNS3	-1.913	0.0014	-1.344	0.134
ILMN_1871457	HS.534680	2.17	0.000151	1.334	0.134
ILMN_1712678	RPS27L	1.722	0.000192	1.229	0.134
ILMN_1687519	SNAP23	1.533	0.000665	1.198	0.135
ILMN_1784447	PLCE1	2.779	0.000267	1.487	0.136
ILMN_2383383	PIR	1.547	0.00068	1.202	0.137
ILMN_1739847	EIF3D	1.423	0.000473	1.153	0.139
ILMN_1660900	SNORA7B	1.475	0.00153	1.191	0.144
ILMN_1740429	FTL	1.615	0.0014	1.234	0.149
ILMN_2324561	SLC7A6	2.577	0.000237	1.42	0.15
ILMN_2111739	MAN2C1	1.771	5.62E-05	1.207	0.151
ILMN_1737124	PRPF4B	1.391	0.0017	1.157	0.153
ILMN_2042771	PTTG1	2.058	0.000433	1.322	0.154
ILMN_1784186	C1ORF170	-2.591	0.000166	-1.398	0.159
ILMN_1675617	NT5M	-1.461	0.00132	-1.175	0.16
ILMN_3194432	LOC100129913	1.667	0.000882	1.231	0.161
ILMN_2412822	SCN3B	1.907	0.000358	1.272	0.162
ILMN_1751431	WIBG	1.446	0.000431	1.15	0.162
ILMN_1721337	MRPS18B	-1.443	0.000639	-1.154	0.164
ILMN_1755047	LRRC2	-2.388	0.00156	-1.45	0.165
ILMN_3200830	LOC649553	1.503	0.000437	1.166	0.165
ILMN_1795826	ATP6V0D1	1.402	0.00055	1.138	0.166
ILMN_1775111	SND1	1.397	0.000961	1.145	0.167
ILMN_1780188	B3GALNT2	-1.393	0.00123	-1.147	0.168
ILMN_1814917	TLE2	-1.812	0.000399	-1.246	0.168
ILMN_1652806	ATP5J	-1.639	0.00109	-1.223	0.169
ILMN_3243961	ZNF252	-1.537	0.0017	-1.2	0.172
ILMN_1711729	LOC442454	-1.302	0.00152	-1.116	0.176
ILMN_1728355	PSMD4	1.448	0.000751	1.153	0.176
ILMN_1815115	CYC1	-1.364	0.000742	-1.127	0.177
ILMN_2136971	FABP3	-2.558	0.000109	-1.358	0.177
ILMN_3305304	POLD2	1.351	0.000731	1.122	0.178

ILMN_1788053	SLC25A12	-1.773	0.00019	-1.214	0.18
ILMN_1770479	LMO7	1.703	0.000255	1.203	0.18
ILMN_1787879	ARL2	1.362	0.000212	1.111	0.181
ILMN_1799024	VAC14	1.501	0.000558	1.163	0.181
ILMN_1653129	CSTF2	1.39	0.00169	1.146	0.182
ILMN_2070072	RPS7	2.046	0.000175	1.27	0.183
ILMN_1805161	LZTR1	1.364	0.00186	1.137	0.186
ILMN_1760741	NDUFA9	-1.573	0.00124	-1.194	0.191
ILMN_1699071	C21ORF7	2.565	0.000491	1.401	0.191
ILMN_2326509	CASP1	1.72	0.000251	1.199	0.195
ILMN_1734867	NR2C1	1.493	0.000661	1.156	0.198
ILMN_1800096	MPST	-1.542	0.000263	-1.154	0.202
ILMN_2199926	OR7E37P	-2.67	0.000317	-1.391	0.202
ILMN_1705224	TMEM110	-1.972	0.00118	-1.289	0.209
ILMN_2384591	HN1	2.223	9.67E-07	1.193	0.21
ILMN_1786308	NIPSNAP3B	-2.243	0.000208	-1.292	0.211
ILMN_1777444	STX5	1.44	0.00129	1.147	0.211
ILMN_1672024	ISCA1L	-1.597	0.00112	-1.188	0.212
ILMN_1758034	ETFDH	-1.71	0.000477	-1.2	0.214
ILMN_2214098	BIVM	-1.442	0.00165	-1.149	0.217
ILMN_3239284	B9D1	1.519	0.00176	1.174	0.217
ILMN_1705297	MYBPH	7.733	0.00129	2.096	0.228
ILMN_1807610	PRPH2	1.83	0.000658	1.226	0.23
ILMN_1815107	MATR3	1.443	0.000826	1.134	0.232
ILMN_3306482	LOC730107	-1.486	0.00191	-1.159	0.233
ILMN_2228732	CCNG2	2.393	0.00139	1.366	0.236
ILMN_1748836	FUZ	-1.375	0.00123	-1.119	0.237
ILMN_2358733	TAZ	-1.361	0.0016	-1.118	0.239
ILMN_1731418	SP110	1.491	0.000875	1.143	0.244
ILMN_1680501	GTF2IRD2B	-2.077	0.000164	-1.234	0.246
ILMN_1786444	LPL	-2.103	0.00182	-1.307	0.246
ILMN_1665132	CD36	-3.09	4.31E-05	-1.335	0.253
ILMN_1669709	TMEM108	-1.788	6.00E-04	-1.202	0.255
ILMN_1750722	RPS7	1.731	0.00187	1.215	0.255
ILMN_1671442	WDR43	1.358	0.00166	1.113	0.257
ILMN_1662917	LMO1	-2.117	0.00124	-1.288	0.259
ILMN_1696087	PHB2	-1.335	0.000933	-1.099	0.261
ILMN_2405521	MTHFD2	2.771	3.76E-05	1.289	0.261
ILMN_2306189	MAGED1	1.726	0.000325	1.175	0.262
ILMN_1656368	ALDH4A1	1.441	0.00183	1.135	0.263
ILMN_2091310	TMEM16A	-1.425	0.000562	-1.115	0.264
ILMN_2201533	C17ORF61	1.437	0.000415	1.115	0.264
ILMN_2234873	NME2	-1.671	0.00109	-1.182	0.269
ILMN_1783333	C16ORF61	-1.607	0.00175	-1.174	0.273
ILMN_2358457	ATF4	-1.547	0.00147	-1.155	0.278
ILMN_2240866	MASP1	-4.5	3.58E-06	-1.364	0.278
ILMN_1814022	NR1H3	1.581	0.000407	1.141	0.281
ILMN_1655311	LOC145853	-2.029	5.16E-06	-1.159	0.283
ILMN_2185563	ANKRA2	1.664	0.000159	1.144	0.286
ILMN_1722239	TIMM8A	-1.689	0.0015	-1.185	0.287
ILMN_1814726	SCARB2	1.416	0.000231	1.098	0.29
ILMN_2262288	EEF1G	-1.431	0.0011	-1.117	0.295
ILMN_1736077	LIAS	-1.553	0.00178	-1.153	0.296
ILMN_1672382	SLC38A3	-2.083	0.000109	-1.202	0.296
ILMN_2399893	RPS24	1.591	0.0014	1.157	0.298
ILMN_1687533	SEMA4D	1.843	0.000718	1.195	0.301
ILMN_2373632	IDH3B	-1.926	0.000497	-1.201	0.305
ILMN_1808501	SH3KBP1	-2.289	0.00107	-1.283	0.305
ILMN_1814589	LOC728037	-1.818	0.000525	-1.181	0.309
ILMN_1806634	NNT	-1.599	0.00104	-1.147	0.319
ILMN_1652797	FAM174B	3.098	0.000116	1.312	0.319

ILMN_1784365	MYOG	2.486	0.000449	1.277	0.32
ILMN_1733176	LIMS1	-1.457	0.000603	-1.108	0.324
ILMN_1691364	STAT1	1.753	0.000319	1.155	0.326
ILMN_1780698	ZFYVE19	-1.356	0.00122	-1.093	0.328
ILMN_1686664	MT2A	3.595	0.000754	1.418	0.335
ILMN_2361768	CHRNA1	7.065	7.50E-05	1.549	0.336
ILMN_1676980	MTSS1	1.608	0.000442	1.129	0.342
ILMN_2043918	DLEU1	-1.787	0.000382	-1.157	0.345
ILMN_1722855	VEGFB	-1.825	2.18E-05	-1.127	0.35
ILMN_1766000	PM20D2	1.607	0.00158	1.144	0.354
ILMN_1740430	SLC2A4RG	-1.366	0.00185	-1.093	0.358
ILMN_1680072	ZNF32	-1.442	0.000354	-1.092	0.36
ILMN_1810782	SH3KBP1	-2.279	0.000792	-1.238	0.362
ILMN_1723494	SIRT2	-1.425	0.00166	-1.102	0.369
ILMN_1775522	MAGED1	1.659	0.000851	1.138	0.371
ILMN_3239735	WASH5P	1.439	0.000469	1.092	0.371
ILMN_1798700	CHRNA1	6.066	0.000113	1.471	0.372
ILMN_2304512	SAA1	15.44	5.02E-05	1.733	0.373
ILMN_1893633	LOC439949	-1.617	0.00125	-1.136	0.374
ILMN_1804662	NRG4	-2.055	0.000237	-1.177	0.375
ILMN_1779751	C7ORF55	-1.537	0.00122	-1.118	0.381
ILMN_1743205	ABCA7	1.656	0.00105	1.135	0.391
ILMN_2052208	GADD45A	3.921	0.000458	1.372	0.391
ILMN_1671402	ARPP-21	2.802	9.00E-04	1.289	0.392
ILMN_1694111	PNKP	1.392	0.000647	1.082	0.395
ILMN_2376289	DBNL	1.72	0.000667	1.137	0.396
ILMN_1768101	HOXB6	-1.742	0.000327	-1.122	0.427
ILMN_1786189	MKI67IP	1.437	0.00159	1.089	0.44
ILMN_2400661	ZNF626	1.479	0.000394	1.083	0.441
ILMN_1686906	TP53INP2	-2.245	0.000789	-1.192	0.443
ILMN_1727740	SYNCRIP	1.451	0.0017	1.091	0.445
ILMN_1722206	MAF	1.576	0.00145	1.109	0.449
ILMN_1690754	SVIL	1.682	0.000353	1.109	0.452
ILMN_1758315	SLC9A9	1.753	0.000369	1.117	0.454
ILMN_3197097	TSTD1	2.163	0.00137	1.182	0.47
ILMN_2148668	RCBTB2	1.721	0.00052	1.111	0.476
ILMN_1672161	ARPP-21	1.983	1.03E-05	1.103	0.478
ILMN_1780298	FAM86A	1.864	0.00071	1.131	0.48
ILMN_1704446	SLC6A10P	-1.599	0.000127	-1.082	0.486
ILMN_1652505	APEX2	1.663	0.00183	1.115	0.49
ILMN_1694075	GADD45A	2.897	0.000864	1.224	0.507
ILMN_1814315	PBXIP1	1.617	3.62E-05	1.07	0.521
ILMN_1798827	SRBD1	1.313	0.00189	-1.055	0.528
ILMN_3269324	FLJ37644	-2.205	0.000361	-1.139	0.531
ILMN_1701514	TRAF3IP2	1.78	0.000785	1.107	0.535
ILMN_1699695	TNFRSF21	1.825	0.00158	1.116	0.547
ILMN_1765409	STAM	1.341	0.00184	-1.056	0.549
ILMN_3234884	KIF22	-1.829	0.00108	-1.108	0.562
ILMN_1775703	TRAPPC6A	1.486	0.00125	1.069	0.568
ILMN_1897333	HS.18849	1.522	0.000264	1.062	0.575
ILMN_1726981	VEGFB	-1.737	0.00017	-1.078	0.581
ILMN_2119224	KIFAP3	1.457	0.000561	1.059	0.581
ILMN_1682147	HOOK2	-1.803	0.000348	-1.089	0.582
ILMN_1751803	LSM10	-1.539	0.00174	-1.075	0.586
ILMN_1728047	AKR1A1	1.912	0.000245	1.093	0.588
ILMN_1676197	LRP11	1.454	0.00129	1.062	0.589
ILMN_1658110	C18ORF19	-1.506	0.000271	-1.057	0.596
ILMN_1662886	KLHL34	-2.945	0.000323	-1.157	0.605
ILMN_2044832	NOP56	1.368	0.00127	1.046	0.631
ILMN_1887174	KIAA0146	1.362	0.00183	1.044	0.652
ILMN_1737344	DDX41	1.337	0.00111	1.039	0.654

ILMN_1696601	VARS	1.581	0.00188	1.06	0.683
ILMN_1674706	MTHFD2	2.349	0.00045	1.096	0.688
ILMN_1671478	CKB	-4.343	2.12E-05	-1.132	0.689
ILMN_1704380	INCA1	-1.469	0.00116	-1.045	0.697
ILMN_1690105	STAT1	1.705	0.000891	1.059	0.709
ILMN_1742358	CA14	-2.773	0.00189	-1.11	0.74
ILMN_1678191	GDF10	-1.658	6.50E-05	-1.038	0.75
ILMN_2289825	ARPP-21	1.866	1.41E-05	1.04	0.758
ILMN_1869087	HS.40289	1.502	0.000566	1.033	0.768
ILMN_1798181	IRF7	2.177	0.000341	1.054	0.798
ILMN_1673409	MGC16121	7.084	2.13E-07	1.074	0.82
ILMN_1674629	C9ORF3	-1.916	0.000138	-1.034	0.832
ILMN_1762093	ABCC12	-3.1	0.000168	-1.056	0.845
ILMN_1800008	ACAT1	-1.53	0.00144	-1.025	0.845
ILMN_2212763	ICAM3	-1.666	0.000394	-1.024	0.862
ILMN_1698673	EFCAB7	3.54	0.00031	1.056	0.867
ILMN_1730523	FAM195A	-1.697	0.000468	-1.019	0.895
ILMN_1764596	MPST	-1.558	3.00E-04	-1.005	0.964
ILMN_2380771	AKR1A1	1.684	0.00156	1	0.999

Supplementary Table 3: Enrichment analysis of 347 differentially expresed genes (downregulated in ICUAW post-ICU day 7 vs controls) at FDR adjusted P-value < 0.05. Gene Ontology (GO) or Human Phenotype (HP) terms with FDR adjusted P-value (hypergeometric test) < 0.05.

Gene Ontology (GO) or Human Phenotype (HP) term	Description	FDR adjusted P-value (hypergeometric test)
GO:0044429	mitochondrial part	2.02E-14
GO:0005743	mitochondrial inner membrane	8.50E-11
GO:0045333	cellular respiration	9.35E-10
GO:0051186	cofactor metabolic process	0.00494
HP:0001941	Acidosis	0.0129
GO:0019752	carboxylic acid metabolic process	0.0134
GO:0035383	thioester metabolic process	0.0137
GO:0001954	positive regulation of cell-matrix adhesion	0.0141
GO:0045244	succinate-CoA ligase complex (GDP-forming)	0.0381
GO:0043183	vascular endothelial growth factor receptor 1 binding	0.0381
GO:0004776	succinate-CoA ligase (GDP-forming) activity	0.0381
GO:0006082	organic acid metabolic process	0.0492

Please note that **Supplementary table 4** is only available as an Excel file.

Supplementary Table 5: Association of module eigengene values with disease status.
Differences in ME expression were tested using a linear mixed effects model to account for correlation between patient samples: ICUAW day 7 vs control and ICUAW month 6 vs control.

Module	Coefficient ICUAW Day 7 vs. control	FDR Adjusted P-Value	Coefficient ICUAW Month 6 vs. control	FDR Adjusted P-Value
1	-2.604E-01	5.770E-05	-9.307E-02	1.791E-01
2	3.067E-01	2.820E-08	9.377E-02	1.127E-01
3	3.983E-02	5.990E-01	1.757E-01	2.805E-02
4	-2.638E-01	3.340E-05	-9.430E-02	1.641E-01
5	5.260E-02	5.118E-01	-4.590E-02	5.920E-01
6	1.906E-01	1.032E-02	1.159E-01	1.435E-01
7	1.836E-01	1.236E-02	5.736E-02	4.627E-01
8	1.397E-01	7.228E-02	5.585E-02	5.019E-01
9	-2.380E-02	7.718E-01	-2.675E-02	7.606E-01
10	1.249E-01	9.043E-02	-5.129E-02	5.156E-01
11	1.736E-01	1.994E-02	1.755E-01	2.589E-02
12	2.446E-01	2.450E-04	9.116E-02	1.955E-01
13	2.092E-01	3.922E-03	1.325E-01	8.747E-02
14	1.923E-01	3.689E-03	-2.564E-02	7.157E-01
15	8.912E-02	2.361E-01	-6.525E-02	4.152E-01
16	-2.312E-01	4.178E-04	-2.067E-01	2.636E-03
17	-2.067E-01	3.918E-03	-7.180E-02	3.491E-01

Supplementary Table 6a. Module 1 Functional Enrichment. Gene Ontology and Human Phenotype categories with FDR < 0.05

Gene Ontology (GO) or Human Phenotype (HP) term	Description	FDR adjusted P-value (hypergeometric test)
GO:0006091	generation of precursor metabolites and energy	2.71E-22
GO:0015980	energy derivation by oxidation of organic compounds	2.56E-18
GO:0030016	myofibril	4.69E-15
GO:0044429	mitochondrial part	5.23E-14
GO:0003012	muscle system process	9.62E-13
HP:0003198	Myopathy	1.34E-10
GO:0005743	mitochondrial inner membrane	2.78E-10
GO:0006006	glucose metabolic process	1.43E-09
GO:0019752	carboxylic acid metabolic process	7.65E-09
GO:0060048	cardiac muscle contraction	8.50E-09
GO:0008307	structural constituent of muscle	9.59E-09
GO:0070252	actin-mediated cell contraction	1.08E-08
GO:0006733	oxidoreduction coenzyme metabolic process	1.40E-08
GO:0009167	purine ribonucleoside monophosphate metabolic process	1.62E-08
GO:0048037	cofactor binding	3.11E-08
HP:0001941	Acidosis	8.29E-08
GO:1990204	oxidoreductase complex	1.11E-07
GO:0046128	purine ribonucleoside metabolic process	1.69E-07
GO:0070469	respiratory chain	6.68E-06
GO:0061061	muscle structure development	8.54E-06
GO:0044712	single-organism catabolic process	1.16E-05
HP:0001644	Dilated cardiomyopathy	1.21E-05
GO:0046496	nicotinamide nucleotide metabolic process	1.49E-05
GO:0042383	sarcolemma	3.32E-05
GO:0016491	oxidoreductase activity	3.88E-05
HP:0002605	Hepatic necrosis	5.77E-05
GO:0019395	fatty acid oxidation	8.65E-05
HP:0003674	Onset	8.68E-05
HP:0011021	Abnormality of circulating enzyme level	0.000114
HP:0004354	Abnormality of carboxylic acid metabolism	0.000131
GO:0006811	ion transport	0.000165
GO:0055008	cardiac muscle tissue morphogenesis	0.000328
HP:0011675	Arrhythmia	0.000374
GO:0042578	phosphoric ester hydrolase activity	0.000448
GO:0016311	dephosphorylation	0.000456
HP:0011804	Abnormality of muscle physiology	0.000494
GO:0055085	transmembrane transport	0.000503
GO:0006744	ubiquinone biosynthetic process	0.000544
GO:0006942	regulation of striated muscle contraction	0.000834
GO:0022892	substrate-specific transporter activity	0.00118
HP:0001635	Congestive heart failure	0.0012
GO:0048878	chemical homeostasis	0.00149
HP:0002171	Gliosis	0.00179
GO:0009055	electron carrier activity	0.0022
HP:0001992	Organic aciduria	0.00228
GO:0055010	ventricular cardiac muscle tissue morphogenesis	0.00266
HP:0001943	Hypoglycemia	0.00276
GO:0014850	response to muscle activity	0.00302
GO:0042805	actinin binding	0.00327
GO:0016459	myosin complex	0.00347
HP:0012103	Abnormality of the mitochondrion	0.00386
GO:0006749	glutathione metabolic process	0.00445
HP:0100578	Lipoatrophy	0.00476
HP:0001325	Hypoglycemic coma	0.00639
HP:0001711	Abnormality of the left ventricle	0.00658
GO:0072709	cellular response to sorbitol	0.00687
GO:0055100	adiponectin binding	0.00687
GO:0031444	slow-twitch skeletal muscle fiber contraction	0.00687
GO:0000309	nicotinamide-nucleotide adenylyltransferase activity	0.00687
GO:0009081	branched-chain amino acid metabolic process	0.00705
GO:0086001	cardiac muscle cell action potential	0.00896

GO:0016769	transferase activity, transferring nitrogenous groups	0.0106
GO:0070069	cytochrome complex	0.014
HP:0000982	Palmoplantar keratoderma	0.0199
HP:0001714	Ventricular hypertrophy	0.0224
GO:0046323	glucose import	0.0226
HP:0003691	Scapular winging	0.0236
GO:0004619	phosphoglycerate mutase activity	0.0262
GO:0004515	nicotinate-nucleotide adenylyltransferase activity	0.0262
GO:0004083	bisphosphoglycerate 2-phosphatase activity	0.0262
GO:0004082	bisphosphoglycerate mutase activity	0.0262
HP:0012438	Abnormal gallbladder physiology	0.027
GO:1901700	response to oxygen-containing compound	0.0315
GO:0016765	transferase activity, transferring alkyl or aryl (other than methyl) grou	0.0348
HP:0000602	Ophthalmoplegia	0.0351
HP:0100022	Abnormality of movement	0.0366
GO:0042803	protein homodimerization activity	0.0369
HP:0002018	Nausea	0.0463
GO:0005516	calmodulin binding	0.0463

Supplementary Table 6b. Module 2 Functional Enrichment. Gene Ontology and Human Phenotype categories w

Gene Ontology (GO) or Human Phenotype (HP) term	Description	FDR adjusted P-value (hypergeometric test)
GO:0044822	poly(A) RNA binding	3.94E-23
GO:0005730	nucleolus	3.48E-17
GO:0042254	ribosome biogenesis	9.62E-13
GO:0034660	ncRNA metabolic process	7.22E-09
GO:0030529	ribonucleoprotein complex	1.79E-07
GO:0044419	interspecies interaction between orgar	3.34E-07
GO:0016032	viral process	4.04E-07
GO:0033365	protein localization to organelle	5.02E-07
GO:0006401	RNA catabolic process	2.34E-06
GO:0000956	nuclear-transcribed mRNA catabolic pr	2.34E-06
GO:0019083	viral transcription	8.48E-06
GO:0006886	intracellular protein transport	1.15E-05
GO:0006612	protein targeting to membrane	3.01E-05
GO:0045047	protein targeting to ER	3.31E-05
GO:0044445	cytosolic part	4.40E-05
GO:0042623	ATPase activity, coupled	0.00029
GO:0006412	translation	0.0015
GO:0000783	nuclear telomere cap complex	0.00172
GO:0048770	pigment granule	0.00187
GO:0010888	negative regulation of lipid storage	0.0024
GO:0046688	response to copper ion	0.004
GO:0009113	purine nucleobase biosynthetic proces	0.00706
GO:0033256	I-kappaB/NF-kappaB complex	0.00878
GO:0030515	snoRNA binding	0.00882
GO:0010875	positive regulation of cholesterol efflu›	0.0124
GO:0010745	negative regulation of macrophage der	0.0124
HP:0002015	Dysphagia	0.014
GO:2000113	negative regulation of cellular macrom	0.0163
GO:0005774	vacuolar membrane	0.0185
GO:0071294	cellular response to zinc ion	0.0202
GO:0071345	cellular response to cytokine stimulus	0.026
HP:0003749	Pelvic girdle muscle weakness	0.027
HP:0003547	Shoulder girdle muscle weakness	0.027
GO:0006974	cellular response to DNA damage stimu	0.0276
GO:0010501	RNA secondary structure unwinding	0.0297
GO:0000049	tRNA binding	0.0297
GO:0071276	cellular response to cadmium ion	0.0313
GO:0042162	telomeric DNA binding	0.0313
GO:0032204	regulation of telomere maintenance	0.0313
GO:0003735	structural constituent of ribosome	0.0343
GO:0010629	negative regulation of gene expression	0.0347
HP:0004377	Hematological neoplasm	0.0404
GO:0045323	interleukin-1 receptor complex	0.0443
GO:0038027	apolipoprotein A-I-mediated signaling		0.0443
GO:0006177	GMP biosynthetic process	0.0443
GO:0015949	nucleobase-containing small molecule	0.0465
HP:0001288	Gait disturbance	0.05

Supplementary Table 6c. Module 3 Functional Enrichment. Gene Ontology and Human Phenotype categories with FDR < 0.05

Gene Ontology (GO) or Human Phenotype (HP) term	Description	FDR adjusted P-value (hypergeometric test)
GO:0005578	proteinaceous extracellular matrix	1.67E-33
GO:0043062	extracellular structure organization	1.36E-32
GO:0005615	extracellular space	1.85E-31
GO:0022610	biological adhesion	8.68E-20
GO:0070161	anchoring junction	2.00E-19
GO:0005509	calcium ion binding	1.40E-18
GO:0042060	wound healing	1.66E-17
GO:2000145	regulation of cell motility	2.64E-16
GO:0007596	blood coagulation	7.52E-15
GO:0001568	blood vessel development	9.05E-15
GO:0032963	collagen metabolic process	1.20E-14
GO:0032403	protein complex binding	3.27E-14
GO:0031252	cell leading edge	7.50E-14
GO:0005581	collagen trimer	4.52E-13
GO:0050839	cell adhesion molecule binding	5.40E-13
GO:0005178	integrin binding	6.90E-13
GO:0000904	cell morphogenesis involved in differentiation	3.52E-12
GO:0022617	extracellular matrix disassembly	3.54E-12
GO:0005539	glycosaminoglycan binding	6.84E-12
GO:0007167	enzyme linked receptor protein signaling pathway	1.57E-11
GO:0030574	collagen catabolic process	2.20E-11
GO:0005201	extracellular matrix structural constituent	3.15E-11
GO:0015629	actin cytoskeleton	6.64E-11
GO:0009986	cell surface	1.16E-10
GO:0030036	actin cytoskeleton organization	2.60E-10
GO:0001525	angiogenesis	2.82E-10
GO:0005788	endoplasmic reticulum lumen	5.45E-10
GO:0030030	cell projection organization	7.93E-09
GO:0030863	cortical cytoskeleton	8.27E-09
GO:0031175	neuron projection development	1.06E-08
GO:0030168	platelet activation	1.12E-08
GO:0001501	skeletal system development	1.15E-08
GO:0048407	platelet-derived growth factor binding	1.16E-08
GO:0006897	endocytosis	2.12E-08
GO:0001503	ossification	2.79E-08
GO:0030864	cortical actin cytoskeleton	3.42E-08
GO:0007229	integrin-mediated signaling pathway	3.71E-08
GO:0008201	heparin binding	4.25E-08
GO:0048585	negative regulation of response to stimulus	4.27E-08
GO:0003779	actin binding	4.30E-08
GO:0016049	cell growth	4.93E-08
GO:0051336	regulation of hydrolase activity	5.56E-08
GO:0042330	taxis	8.22E-08
GO:0022603	regulation of anatomical structure morphogenesis	1.58E-07
GO:0001101	response to acid chemical	2.02E-07
HP:0000006	Autosomal dominant inheritance	6.52E-07
GO:0045121	membrane raft	7.13E-07
GO:0030193	regulation of blood coagulation	1.24E-06
GO:0032835	glomerulus development	1.34E-06
GO:0070848	response to growth factor	1.36E-06
GO:0051241	negative regulation of multicellular organismal process	1.52E-06
GO:0009887	organ morphogenesis	2.65E-06
HP:0000541	Retinal detachment	4.93E-06
HP:0001388	Joint laxity	5.14E-06
GO:0061134	peptidase regulator activity	5.62E-06
HP:0005111	Dilatation of the ascending aorta	6.62E-06
GO:0050840	extracellular matrix binding	7.01E-06
HP:0000015	Bladder diverticulum	1.11E-05
HP:0002645	Wormian bones	1.28E-05

GO:0009968	negative regulation of signal transduction	1.79E-05
GO:0046903	secretion	1.84E-05
HP:0000938	Osteopenia	2.11E-05
GO:0061448	connective tissue development	2.32E-05
GO:0008285	negative regulation of cell proliferation	2.77E-05
GO:0009897	external side of plasma membrane	3.03E-05
GO:0044853	plasma membrane raft	3.67E-05
HP:0001892	Abnormal bleeding	3.86E-05
GO:0051345	positive regulation of hydrolase activity	4.13E-05
GO:0031091	platelet alpha granule	4.53E-05
GO:0006027	glycosaminoglycan catabolic process	4.53E-05
GO:0071495	cellular response to endogenous stimulus	4.61E-05
GO:0051130	positive regulation of cellular component organization	5.87E-05
HP:0002797	Osteolysis	5.91E-05
GO:0005044	scavenger receptor activity	6.32E-05
HP:0001788	Premature rupture of membranes	7.49E-05
GO:0048771	tissue remodeling	8.29E-05
GO:0044273	sulfur compound catabolic process	8.59E-05
GO:0031226	intrinsic component of plasma membrane	8.86E-05
GO:0009967	positive regulation of signal transduction	9.80E-05
GO:0007264	small GTPase mediated signal transduction	0.000103
GO:0071230	cellular response to amino acid stimulus	0.00011
GO:0005796	Golgi lumen	0.00012
GO:0070208	protein heterotrimerization	0.000126
GO:0009415	response to water	0.000135
HP:0000977	Soft skin	0.000137
HP:0001073	Cigarette-paper scars	0.000175
GO:0060284	regulation of cell development	0.000176
HP:0001633	Abnormality of the mitral valve	0.000187
GO:0052547	regulation of peptidase activity	0.000195
GO:0072376	protein activation cascade	0.000205
GO:0051093	negative regulation of developmental process	0.000242
HP:0004415	Pulmonary artery stenosis	0.000315
GO:0060485	mesenchyme development	0.000315
HP:0003549	Abnormality of connective tissue	0.000323
HP:0000023	Inguinal hernia	0.000328
HP:0000934	Chondrocalcinosis	0.000342
GO:0035987	endodermal cell differentiation	0.000342
GO:2000394	positive regulation of lamellipodium morphogenesis	0.000392
GO:0006024	glycosaminoglycan biosynthetic process	0.000414
HP:0007502	Follicular hyperkeratosis	0.000507
HP:0000703	Dentinogenesis imperfecta	0.000507
HP:0005222	Bowel diverticulosis	0.000557
GO:0061073	ciliary body morphogenesis	0.000679
GO:0007417	central nervous system development	0.000753
GO:0034329	cell junction assembly	0.000812
HP:0006460	Increased laxity of ankles	0.000879
HP:0006316	Irregularly spaced teeth	0.000879
HP:0002010	Narrow maxilla	0.000879
HP:0002982	Tibial bowing	0.000923
GO:0005543	phospholipid binding	0.000967
GO:0002020	protease binding	0.00109
GO:0060429	epithelium development	0.00117
GO:0006873	cellular ion homeostasis	0.00122
GO:0001837	epithelial to mesenchymal transition	0.00142
HP:0004331	Decreased skull ossification	0.00143
GO:0001968	fibronectin binding	0.0015
GO:0060322	head development	0.00155
GO:0031093	platelet alpha granule lumen	0.00155
GO:0045807	positive regulation of endocytosis	0.00161
GO:0031253	cell projection membrane	0.00169
GO:0050673	epithelial cell proliferation	0.00189
GO:0044091	membrane biogenesis	0.00199
GO:0007009	plasma membrane organization	0.00199
GO:0019904	protein domain specific binding	0.00242
GO:0031579	membrane raft organization	0.00243

GO:0042383	sarcolemma	0.00249
GO:0031947	negative regulation of glucocorticoid biosynthetic proce	0.00263
GO:0031944	negative regulation of glucocorticoid metabolic process	0.00263
GO:0005017	platelet-derived growth factor-activated receptor activit	0.00263
GO:0070613	regulation of protein processing	0.00278
HP:0000765	Abnormality of the thorax	0.00298
GO:0030898	actin-dependent ATPase activity	0.00298
GO:0043202	lysosomal lumen	0.00299
HP:0002579	Gastrointestinal dysmotility	0.00315
GO:0038096	Fc-gamma receptor signaling pathway involved in phagc	0.00339
HP:0005988	Congenital muscular torticollis	0.00341
HP:0005758	Basilar impression	0.00341
GO:0002682	regulation of immune system process	0.00354
GO:0048306	calcium-dependent protein binding	0.00372
GO:0071295	cellular response to vitamin	0.00468
GO:0048552	regulation of metalloenzyme activity	0.0048
GO:0005516	calmodulin binding	0.0048
GO:0018108	peptidyl-tyrosine phosphorylation	0.00491
GO:0090132	epithelium migration	0.00495
HP:0012372	Abnormal eye morphology	0.00527
GO:0006954	inflammatory response	0.00544
GO:0060401	cytosolic calcium ion transport	0.00571
GO:0050730	regulation of peptidyl-tyrosine phosphorylation	0.00605
GO:0045177	apical part of cell	0.00618
GO:0043197	dendritic spine	0.00618
GO:0019798	procollagen-proline dioxygenase activity	0.00638
GO:0030659	cytoplasmic vesicle membrane	0.00644
HP:0000325	Triangular face	0.00767
GO:0007423	sensory organ development	0.00767
GO:0043065	positive regulation of apoptotic process	0.00787
GO:0048745	smooth muscle tissue development	0.00822
HP:0005855	Multiple prenatal fractures	0.00826
HP:0003713	Muscle fiber necrosis	0.00826
HP:0002877	Nocturnal hypoventilation	0.00826
GO:0008284	positive regulation of cell proliferation	0.0083
HP:0000309	Abnormality of the midface	0.00851
HP:0001083	Ectopia lentis	0.00856
GO:2000377	regulation of reactive oxygen species metabolic process	0.00888
GO:0007204	positive regulation of cytosolic calcium ion concentratio	0.0094
GO:0048729	tissue morphogenesis	0.00998
GO:0048608	reproductive structure development	0.0104
GO:0061061	muscle structure development	0.0107
GO:0070482	response to oxygen levels	0.0115
HP:0000926	Platyspondyly	0.0117
HP:0008873	Disproportionate short-limb short stature	0.0121
HP:0000520	Proptosis	0.0122
GO:0072007	mesangial cell differentiation	0.0124
GO:0019898	extrinsic component of membrane	0.0132
GO:0060765	regulation of androgen receptor signaling pathway	0.0134
GO:0021782	glial cell development	0.0134
HP:0001650	Aortic valve stenosis	0.0137
HP:0002586	Peritonitis	0.016
HP:0000655	Vitreoretinal degeneration	0.016
HP:0000394	Lop ear	0.016
GO:1903923	positive regulation of protein processing in phagocytic v	0.0165
GO:1902396	protein localization to tight junction	0.0165
GO:0086098	angiotensin-activated signaling pathway involved in hea	0.0165
GO:0034684	integrin alphav-beta5 complex	0.0165
GO:0014846	esophagus smooth muscle contraction	0.0165
GO:0008459	chondroitin 6-sulfotransferase activity	0.0165
GO:0001868	regulation of complement activation, lectin pathway	0.0165
GO:0090218	positive regulation of lipid kinase activity	0.0168
GO:0043531	ADP binding	0.0168
GO:0044089	positive regulation of cellular component biogenesis	0.0181
HP:0000272	Malar flattening	0.0186
GO:0043567	regulation of insulin-like growth factor receptor signalin	0.0201

GO:0030674	protein binding, bridging	0.0203
GO:1900040	regulation of interleukin-2 secretion	0.021
GO:0072071	kidney interstitial fibroblast differentiation	0.021
GO:0046325	negative regulation of glucose import	0.021
GO:0003184	pulmonary valve morphogenesis	0.021
HP:0008780	Congenital bilateral hip dislocation	0.0213
HP:0006067	Multiple carpal ossification centers	0.0213
HP:0005897	Severe osteoporosis	0.0213
HP:0002691	Platybasia	0.0213
HP:0001757	High-frequency sensorineural hearing impairment	0.0213
HP:0001149	Lattice corneal dystrophy	0.0213
HP:0000362	Otosclerosis	0.0213
GO:2001236	regulation of extrinsic apoptotic signaling pathway	0.0236
HP:0005750	Contractures of the joints of the lower limbs	0.025
HP:0000963	Thin skin	0.0252
HP:0003741	Congenital muscular dystrophy	0.026
GO:0051960	regulation of nervous system development	0.026
GO:2000811	negative regulation of anoikis	0.0265
HP:0002960	Autoimmunity	0.0281
GO:0010562	positive regulation of phosphorus metabolic process	0.0283
HP:0002996	Limited elbow movement	0.0288
GO:0006029	proteoglycan metabolic process	0.0293
GO:0048534	hematopoietic or lymphoid organ development	0.0301
HP:0003077	Hyperlipidemia	0.0304
GO:0070374	positive regulation of ERK1 and ERK2 cascade	0.0323
GO:0098739	import across plasma membrane	0.0325
GO:0061314	Notch signaling involved in heart development	0.0325
GO:0032060	bleb assembly	0.0325
GO:0021670	lateral ventricle development	0.0325
GO:0017187	peptidyl-glutamic acid carboxylation	0.0325
GO:0005218	intracellular ligand-gated calcium channel activity	0.0325
GO:0004065	arylsulfatase activity	0.0325
HP:0001844	Abnormality of the hallux	0.0327
HP:0000260	Wide anterior fontanel	0.0328
HP:0008180	Mildly elevated creatine phosphokinase	0.0343
HP:0000473	Torticollis	0.0343
GO:0051258	protein polymerization	0.0351
GO:0030246	carbohydrate binding	0.0358
GO:0045428	regulation of nitric oxide biosynthetic process	0.0366
GO:0007568	aging	0.0371
GO:0043235	receptor complex	0.0412
HP:0001799	Short nail	0.0421
HP:0001533	Slender build	0.0421
GO:0003094	glomerular filtration	0.0432
HP:0002715	Abnormality of the immune system	0.044
GO:0030175	filopodium	0.0451
HP:0000545	Myopia	0.0457
GO:0019216	regulation of lipid metabolic process	0.0457
GO:0097284	hepatocyte apoptotic process	0.0473
GO:0035313	wound healing, spreading of epidermal cells	0.0473
GO:1902174	positive regulation of keratinocyte apoptotic process	0.0481
GO:0097513	myosin II filament	0.0481
GO:0048251	elastic fiber assembly	0.0481
GO:0008449	N-acetylglucosamine-6-sulfatase activity	0.0481
GO:0007181	transforming growth factor beta receptor complex asser	0.0481
GO:0005220	inositol 1,4,5-trisphosphate-sensitive calcium-release ch	0.0481
GO:0004992	platelet activating factor receptor activity	0.0481
GO:0004528	phosphodiesterase I activity	0.0481
GO:0002826	negative regulation of T-helper 1 type immune response	0.0481
GO:0004252	serine-type endopeptidase activity	0.05

Supplementary Table 6d. Module 4 Functional Enrichment. Gene Ontology and Human Phenotype categories with FDR < 0.05

Gene Ontology (GO) or Human Phenotype (HP) term	Description	FDR adjusted P-value (hypergeometric test)
GO:0044429	mitochondrial part	2.04E-59
GO:0005743	mitochondrial inner membrane	4.08E-53
GO:0070124	mitochondrial translational initiation	6.90E-28
GO:0070126	mitochondrial translational termination	3.24E-25
GO:0022904	respiratory electron transport chain	4.95E-24
GO:0070469	respiratory chain	1.36E-19
GO:0009205	purine ribonucleoside triphosphate metabolic process	3.43E-19
GO:0046034	ATP metabolic process	4.12E-19
GO:0000313	organellar ribosome	3.67E-18
GO:0050136	NADH dehydrogenase (quinone) activity	1.16E-13
GO:0030964	NADH dehydrogenase complex	7.29E-12
GO:0005753	mitochondrial proton-transporting ATP synthase complex	2.55E-11
GO:0003735	structural constituent of ribosome	3.05E-11
GO:1990542	mitochondrial transmembrane transport	3.23E-10
HP:0004360	Abnormality of acid-base homeostasis	3.18E-09
GO:0015078	hydrogen ion transmembrane transporter activity	4.76E-09
GO:1902600	hydrogen ion transmembrane transport	1.40E-08
HP:0001254	Lethargy	8.58E-06
HP:0003546	Exercise intolerance	0.000141
HP:0001298	Encephalopathy	0.000178
HP:0002490	Increased CSF lactate	0.000232
HP:0002878	Respiratory failure	0.000253
HP:0001427	Mitochondrial inheritance	0.000268
HP:0003287	Abnormality of mitochondrial metabolism	0.000435
HP:0004481	Progressive macrocephaly	0.000597
GO:0018065	protein-cofactor linkage	0.00101
HP:0000543	Optic disc pallor	0.00203
GO:0070585	protein localization to mitochondrion	0.00238
GO:0051536	iron-sulfur cluster binding	0.00301
GO:0016226	iron-sulfur cluster assembly	0.00324
GO:0004129	cytochrome-c oxidase activity	0.00603
HP:0002181	Cerebral edema	0.00686
HP:0001639	Hypertrophic cardiomyopathy	0.0108
HP:0002415	Leukodystrophy	0.0165
GO:0032981	mitochondrial respiratory chain complex I assembly	0.0242
GO:1990617	CHOP-ATF4 complex	0.0307
GO:0016838	carbon-oxygen lyase activity, acting on phosphates	0.0307
HP:0001423	X-linked dominant inheritance	0.0427
HP:0000618	Blindness	0.0432
GO:0043522	leucine zipper domain binding	0.0487
HP:0001133	Constricted visual fields	0.05

Supplementary Table 6e. Module 6 Functional Enrichment. Gene Ontology and Human Phenotype categories with FDR < 0.05

Gene Ontology (GO) or Human Phenotype (HP) term	Description	FDR adjusted P-value (hypergeometric test)
GO:0016023	cytoplasmic membrane-bounded vesicle	5.82E-06
GO:0040012	regulation of locomotion	1.86E-05
GO:0036500	ATF6-mediated unfolded protein response	5.80E-05
GO:0006897	endocytosis	6.96E-05
GO:0045121	membrane raft	7.81E-05
GO:0070161	anchoring junction	0.000364
GO:2000145	regulation of cell motility	0.000366
GO:0034975	protein folding in endoplasmic reticulum	0.000429
GO:0098602	single organism cell adhesion	0.00044
GO:0002699	positive regulation of immune effector process	0.000477
GO:0046718	viral entry into host cell	0.000946
GO:0030659	cytoplasmic vesicle membrane	0.00101
HP:0000095	Abnormality of the glomerulus	0.00142
GO:0034097	response to cytokine	0.00163
HP:0012379	Abnormal enzyme/coenzyme activity	0.00209
GO:0007264	small GTPase mediated signal transduction	0.00291
GO:0032270	positive regulation of cellular protein metabolic	0.00293
GO:0050839	cell adhesion molecule binding	0.00338
GO:0010008	endosome membrane	0.00364
GO:0098552	side of membrane	0.00375
HP:0005339	Abnormality of complement system	0.00383
GO:0050211	procollagen galactosyltransferase activity	0.00393
GO:0005788	endoplasmic reticulum lumen	0.00487
HP:0002756	Pathologic fracture	0.00647
GO:0009986	cell surface	0.00685
GO:0007568	aging	0.00696
GO:0005178	integrin binding	0.00866
HP:0002645	Wormian bones	0.00874
GO:0006098	pentose-phosphate shunt	0.00891
HP:0000467	Neck muscle weakness	0.01
HP:0008944	Distal lower limb amyotrophy	0.01
HP:0000418	Narrow nasal ridge	0.01
HP:0005474	Decreased calvarial ossification	0.01
GO:0032880	regulation of protein localization	0.0102
HP:0000100	Nephrotic syndrome	0.0111
HP:0002703	Abnormality of skull ossification	0.0114
GO:0042270	protection from natural killer cell mediated cyto	0.0117
GO:0010562	positive regulation of phosphorus metabolic pro	0.012
GO:0031625	ubiquitin protein ligase binding	0.0139
HP:0007394	Prominent superficial blood vessels	0.0139
HP:0000455	Broad nasal tip	0.0146
GO:0031399	regulation of protein modification process	0.0148
HP:0000248	Brachycephaly	0.016
HP:0000520	Proptosis	0.017
HP:0000774	Narrow chest	0.0172
GO:0042269	regulation of natural killer cell mediated cytotox	0.018
HP:0004944	Cerebral aneurysm	0.0184
HP:0000764	Peripheral axonal degeneration	0.0184
HP:0002754	Osteomyelitis	0.0225
GO:0019322	pentose biosynthetic process	0.0232
GO:0042296	ISG15 transferase activity	0.0232
GO:0004619	phosphoglycerate mutase activity	0.0232
GO:0004083	bisphosphoglycerate 2-phosphatase activity	0.0232
GO:0004082	bisphosphoglycerate mutase activity	0.0232
GO:0048475	coated membrane	0.0235
HP:0002185	Neurofibrillary tangles	0.0235
HP:0001744	Splenomegaly	0.0246
GO:0045087	innate immune response	0.0253

GO:0071363	cellular response to growth factor stimulus	0.0272
GO:0051129	negative regulation of cellular component organ	0.0297
HP:0001284	Areflexia	0.0307
HP:0009771	Osteolytic defects of the phalanges of the hand	0.0354
HP:0002813	Abnormality of limb bone morphology	0.0376
GO:0007229	integrin-mediated signaling pathway	0.0402
GO:0097190	apoptotic signaling pathway	0.0414
HP:0000605	Supranuclear gaze palsy	0.0421
GO:0048585	negative regulation of response to stimulus	0.0481
HP:0002679	Abnormality of the sella turcica	0.0494
GO:0042149	cellular response to glucose starvation	0.05
CORUM:1223	H2AX complex, isolated from cells without IR ex[illegible]	0.05

Supplementary Table 6f. Module 11 Functional Enrichment. Gene Ontology and Human Phenotype categories with FDR < 0.05

Gene Ontology (GO) or Human Phenotype (HP) term	Description	FDR adjusted P-value (hypergeometric test)
GO:0006954	inflammatory response	2.41E-08
GO:0050776	regulation of immune response	2.95E-08
GO:0045087	innate immune response	3.00E-08
GO:0001816	cytokine production	4.52E-08
GO:0030334	regulation of cell migration	4.21E-07
GO:0002252	immune effector process	7.36E-07
GO:1903557	positive regulation of tumor necrosis factor su	6.84E-06
GO:0006909	phagocytosis	1.48E-05
GO:0002274	myeloid leukocyte activation	1.50E-05
GO:0014068	positive regulation of phosphatidylinositol 3-k	1.58E-05
GO:0030595	leukocyte chemotaxis	1.69E-05
GO:0006911	phagocytosis, engulfment	1.92E-05
HP:0005356	Decreased serum complement factor I	2.34E-05
GO:0072376	protein activation cascade	3.96E-05
HP:0000793	Membranoproliferative glomerulonephritis	5.82E-05
GO:0002573	myeloid leukocyte differentiation	6.74E-05
GO:0097190	apoptotic signaling pathway	9.61E-05
GO:0030036	actin cytoskeleton organization	0.000103
GO:0008360	regulation of cell shape	0.000108
GO:0005602	complement component C1 complex	0.000182
GO:0002250	adaptive immune response	0.000191
GO:0006956	complement activation	0.000216
GO:0070661	leukocyte proliferation	0.000269
GO:0045807	positive regulation of endocytosis	0.000292
GO:0046427	positive regulation of JAK-STAT cascade	0.000338
GO:0030041	actin filament polymerization	0.000352
GO:0030155	regulation of cell adhesion	0.000643
HP:0002725	Systemic lupus erythematosus	0.000685
GO:0050715	positive regulation of cytokine secretion	0.000786
GO:0007169	transmembrane receptor protein tyrosine kin	0.00101
GO:0051247	positive regulation of protein metabolic proce	0.00102
GO:0043552	positive regulation of phosphatidylinositol 3-k	0.00105
GO:0031347	regulation of defense response	0.00107
HP:0200098	Absent skin pigmentation	0.00108
HP:0007544	Piebaldism	0.00108
GO:0097169	AIM2 inflammasome complex	0.00108
GO:0009617	response to bacterium	0.00115
GO:0006775	fat-soluble vitamin metabolic process	0.00122
GO:0071222	cellular response to lipopolysaccharide	0.00133
GO:0009986	cell surface	0.00137
GO:0072559	NLRP3 inflammasome complex	0.0018
GO:0042327	positive regulation of phosphorylation	0.00196
GO:0030833	regulation of actin filament polymerization	0.00199
GO:0043303	mast cell degranulation	0.00234
GO:0032762	mast cell cytokine production	0.00269
GO:0016892	endoribonuclease activity, producing 3'-phosp	0.00269
GO:0009611	response to wounding	0.00269
GO:0042531	positive regulation of tyrosine phosphorylatio	0.00293
GO:0005773	vacuole	0.00392
GO:0007229	integrin-mediated signaling pathway	0.00408
GO:0031529	ruffle organization	0.00436
GO:0042362	fat-soluble vitamin biosynthetic process	0.00498
GO:0043085	positive regulation of catalytic activity	0.00596
GO:0031953	negative regulation of protein autophosphory	0.00638
HP:0007443	Partial albinism	0.00644
GO:0038024	cargo receptor activity	0.00724
GO:0019369	arachidonic acid metabolic process	0.00781
GO:0044130	negative regulation of growth of symbiont in l	0.00794
GO:0048011	neurotrophin TRK receptor signaling pathway	0.00894
GO:0034097	response to cytokine	0.0113

GO:0005092	GDP-dissociation inhibitor activity	0.0116
GO:0043069	negative regulation of programmed cell death	0.0123
GO:0032103	positive regulation of response to external sti	0.0124
GO:0048872	homeostasis of number of cells	0.0141
GO:0001891	phagocytic cup	0.0158
HP:0100523	Liver abscess	0.016
GO:0051241	negative regulation of multicellular organisma	0.0161
HP:0001025	Urticaria	0.0174
GO:0042802	identical protein binding	0.0174
GO:0002675	positive regulation of acute inflammatory resp	0.0182
GO:0019902	phosphatase binding	0.0189
GO:0043372	positive regulation of CD4-positive, alpha-beta	0.0207
GO:0016505	peptidase activator activity involved in apopto	0.0207
HP:0002211	White forelock	0.0223
HP:0000727	Frontal lobe dementia	0.0223
GO:0043065	positive regulation of apoptotic process	0.0243
GO:1903409	reactive oxygen species biosynthetic process	0.0256
GO:0004540	ribonuclease activity	0.0256
HP:0000421	Epistaxis	0.031
GO:0030141	secretory granule	0.0311
GO:0005581	collagen trimer	0.0322
GO:1990682	CSF1-CSF1R complex	0.033
GO:1990246	uniplex complex	0.033
GO:1900387	negative regulation of cell-cell adhesion by ne	0.033
GO:0071718	sodium-independent icosanoid transport	0.033
GO:0070976	TIR domain binding	0.033
GO:0051022	Rho GDP-dissociation inhibitor binding	0.033
GO:0050783	cocaine metabolic process	0.033
GO:0043376	regulation of CD8-positive, alpha-beta T cell d	0.033
GO:0038162	erythropoietin-mediated signaling pathway	0.033
GO:0035921	desmosome disassembly	0.033
GO:0035509	negative regulation of myosin-light-chain-pho	0.033
GO:0034988	Fc-gamma receptor I complex binding	0.033
GO:0030421	defecation	0.033
GO:0017024	myosin I binding	0.033
GO:0014739	positive regulation of muscle hyperplasia	0.033
GO:0014016	neuroblast differentiation	0.033
GO:0005121	Toll binding	0.033
GO:0005020	stem cell factor receptor activity	0.033
GO:0005011	macrophage colony-stimulating factor recepto	0.033
GO:0004796	thromboxane-A synthase activity	0.033
GO:0002930	trabecular meshwork development	0.033
GO:1900026	positive regulation of substrate adhesion-dep	0.0356
GO:0045730	respiratory burst	0.0356
GO:0050795	regulation of behavior	0.0363
GO:0030027	lamellipodium	0.0383
GO:0090501	RNA phosphodiester bond hydrolysis	0.0397
GO:0002431	Fc receptor mediated stimulatory signaling pa	0.0413
GO:0060071	Wnt signaling pathway, planar cell polarity pa	0.0426
GO:0031225	anchored component of membrane	0.0464
HP:0002354	Memory impairment	0.05

Supplementary Table 6g. Module 17 Functional Enrichment. Gene Ontology and Human Phenotype categories with FDR < 0.05

Gene Ontology (GO) or Human Phenotype (HP) term	Description	FDR adjusted P-value (hypergeometric test)
GO:0035295	tube development	6.40E-10
GO:0001525	angiogenesis	2.07E-09
GO:0048568	embryonic organ development	2.52E-08
GO:0003007	heart morphogenesis	3.07E-08
GO:0016477	cell migration	2.08E-07
GO:0090132	epithelium migration	2.54E-07
GO:0009855	determination of bilateral symmetry	8.60E-07
GO:0001947	heart looping	1.18E-06
GO:0045165	cell fate commitment	0.000107
GO:0001822	kidney development	0.000301
HP:0001901	Polycythemia	0.000371
GO:0007386	compartment pattern specification	0.000473
GO:0001666	response to hypoxia	0.000483
GO:0030324	lung development	0.000682
GO:0048738	cardiac muscle tissue development	0.000781
GO:0045668	negative regulation of osteoblast differentiation	0.000796
GO:0001974	blood vessel remodeling	0.000796
GO:0050673	epithelial cell proliferation	0.000802
GO:0045765	regulation of angiogenesis	0.000951
GO:0021700	developmental maturation	0.00122
GO:0048663	neuron fate commitment	0.00131
GO:1903707	negative regulation of hemopoiesis	0.00142
GO:0007155	cell adhesion	0.00153
GO:0040012	regulation of locomotion	0.00154
GO:0033002	muscle cell proliferation	0.00263
GO:0048711	positive regulation of astrocyte differentiation	0.00281
HP:0004936	Venous thrombosis	0.0029
GO:0031226	intrinsic component of plasma membrane	0.00338
GO:0009611	response to wounding	0.00346
GO:0070986	left/right axis specification	0.0035
GO:0030334	regulation of cell migration	0.00363
GO:0048715	negative regulation of oligodendrocyte differentiation	0.00427
HP:0002624	Venous abnormality	0.00456
GO:0051094	positive regulation of developmental process	0.00498
GO:0051240	positive regulation of multicellular organismal process	0.00537
GO:0019199	transmembrane receptor protein kinase activity	0.0063
GO:0045649	regulation of macrophage differentiation	0.00702
GO:0030900	forebrain development	0.00713
GO:0008285	negative regulation of cell proliferation	0.00729
GO:0009967	positive regulation of signal transduction	0.00774
GO:0097150	neuronal stem cell maintenance	0.00808
GO:0006182	cGMP biosynthetic process	0.00808
GO:0061138	morphogenesis of a branching epithelium	0.00922
GO:0048863	stem cell differentiation	0.0119
GO:0000122	negative regulation of transcription from RNA polymerase II prc	0.0119
GO:0061384	heart trabecula morphogenesis	0.013
GO:0007220	Notch receptor processing	0.013
GO:0005912	adherens junction	0.0139
GO:0048545	response to steroid hormone	0.0143
GO:0032102	negative regulation of response to external stimulus	0.0143
GO:0042491	auditory receptor cell differentiation	0.0145
GO:0008284	positive regulation of cell proliferation	0.0146
HP:0011029	Internal hemorrhage	0.0151
GO:0045121	membrane raft	0.0164
GO:0048566	embryonic digestive tract development	0.0175
GO:0046875	ephrin receptor binding	0.0175
HP:0002040	Esophageal varix	0.018
GO:1901222	regulation of NIK/NF-kappaB signaling	0.0191
GO:0045777	positive regulation of blood pressure	0.0191

GO:0009925	basal plasma membrane	0.0191
GO:0030513	positive regulation of BMP signaling pathway	0.0208
GO:0060412	ventricular septum morphogenesis	0.0226
GO:0045604	regulation of epidermal cell differentiation	0.0226
GO:2001200	positive regulation of dendritic cell differentiation	0.0237
GO:2000637	positive regulation of gene silencing by miRNA	0.0237
GO:0085018	maintenance of symbiont-containing vacuole by host	0.0237
GO:0071931	positive regulation of transcription involved in G1/S transition c	0.0237
GO:0071288	cellular response to mercury ion	0.0237
GO:0060843	venous endothelial cell differentiation	0.0237
GO:0046878	positive regulation of saliva secretion	0.0237
GO:0035379	carbon dioxide transmembrane transporter activity	0.0237
GO:0035378	carbon dioxide transmembrane transport	0.0237
GO:0035377	transepithelial water transport	0.0237
GO:0030184	nitric oxide transmembrane transporter activity	0.0237
GO:0003270	Notch signaling pathway involved in regulation of secondary he	0.0237
GO:0043066	negative regulation of apoptotic process	0.0287
GO:0050999	regulation of nitric-oxide synthase activity	0.0303
GO:1903507	negative regulation of nucleic acid-templated transcription	0.0316
HP:0001342	Cerebral hemorrhage	0.0361
HP:0001048	Cavernous hemangioma	0.0416
GO:0045600	positive regulation of fat cell differentiation	0.0439
GO:0043392	negative regulation of DNA binding	0.0463
GO:2001027	negative regulation of endothelial cell chemotaxis	0.0474
GO:1902263	apoptotic process involved in embryonic digit morphogenesis	0.0474
GO:0098507	polynucleotide 5' dephosphorylation	0.0474
GO:0090164	asymmetric Golgi ribbon formation	0.0474
GO:0072563	endothelial microparticle	0.0474
GO:0061030	epithelial cell differentiation involved in mammary gland alveol	0.0474
GO:0060352	cell adhesion molecule production	0.0474
GO:0048866	stem cell fate specification	0.0474
GO:0036273	response to statin	0.0474
GO:0035799	ureter maturation	0.0474
GO:0035331	negative regulation of hippo signaling	0.0474
GO:0034617	tetrahydrobiopterin binding	0.0474
GO:0030950	establishment or maintenance of actin cytoskeleton polarity	0.0474
GO:0021894	cerebral cortex GABAergic interneuron development	0.0474
GO:0021830	interneuron migration from the subpallium to the cortex	0.0474
GO:0020003	symbiont-containing vacuole	0.0474
GO:0018262	isopeptide cross-linking	0.0474
GO:0014806	smooth muscle hyperplasia	0.0474
GO:0014740	negative regulation of muscle hyperplasia	0.0474
GO:0004651	polynucleotide 5'-phosphatase activity	0.0474
GO:0004517	nitric-oxide synthase activity	0.0474
GO:0003192	mitral valve formation	0.0474
GO:0001300	chronological cell aging	0.0474
GO:0007163	establishment or maintenance of cell polarity	0.0488
GO:0007167	enzyme linked receptor protein signaling pathway	0.0497

Supplemental Table 7a: Module 1 transcription factor binding site (TFBS) overrepresentation analysis.
Results with Fisher score >= 7 and Z-score >= 10 shown

TFBS	JASPAR ID	Z-score	Fisher score
MEF2A	MA0052.1	14.385	20.368
RORA_1	MA0071.1	12.51	17.763
NR4A2	MA0160.1	10.25	15.888
Esrrb	MA0141.1	11.952	15.139
HNF4A	MA0114.1	10.266	9.604

Supplemental Table 7b: Module 2 transcription factor binding site (TFBS) overrepresentation analysis.

Results with Fisher score >= 7 and Z-score >= 10 shown

TFBS	JASPAR ID	Z-score	Fisher score
GABPA	MA0062.2	11.195	9.003

Supplemental Data 7c: mod 3 transcription factor binding site (TFBS) overrepresentation analysis.

Supplemental Table 7c: Module 3 transcription factor binding site (TFBS) overrepresentation analysis.

Results with Fisher score >= 7 and Z-score >= 10 shown

TFBS	JASPAR ID	Z-score	Fisher score
TEAD1	MA0090.1	16.06	20.748
HOXA5	MA0158.1	28.527	18.847
SPI1	MA0080.2	14.121	18.634
RUNX1	MA0002.2	21.493	18.143
NFATC2	MA0152.1	23.135	17.052
STAT1	MA0137.2	15.012	16.347
FEV	MA0156.1	14.803	16.091
CEBPA	MA0102.2	18.63	15.788
FOXO3	MA0157.1	19.586	15.289
ELF5	MA0136.1	20.4	15.006
SRY	MA0084.1	17.844	14.994
SOX9	MA0077.1	16.859	14.378
Foxa2	MA0047.2	17.228	14.11
FOXA1	MA0148.1	25.226	14.013
Prrx2	MA0075.1	14.274	13.669
Gfi	MA0038.1	18.076	13.66
Sox5	MA0087.1	12.409	12.761
Pdx1	MA0132.1	19.284	12.534
Nobox	MA0125.1	18.448	12.222
SPIB	MA0081.1	14.152	12.132

Hand1::Tcfe2a	MA0092.1	17.801	12.028
FOXI1	MA0042.1	16.889	11.279
FOXD1	MA0031.1	16.83	10.767
Foxd3	MA0041.1	16.79	10.706
ARID3A	MA0151.1	15.298	10.561
AP1	MA0099.2	13.534	10.21
Sox17	MA0078.1	15.28	8.818
Nkx2-5	MA0063.1	18.068	8.666
Sox2	MA0143.1	10.609	8.145
IRF1	MA0050.1	11.417	7.737
Ddit3::Cebpa	MA0019.1	12.315	7.574
TAL1::TCF3	MA0091.1	12.704	7.492

Supplemental Table 7d: Module 6 transcription factor binding site (TFBS) overrepresentation analysis.

Results with Fisher score >= 7 and Z-score >= 10 shown

TFBS	JASPAR ID	Z-score	Fisher score
ELK1	MA0028.1	11.17	7.803

Supplemental Table 7e: Module 7 transcription factor binding site (TFBS) overrepresentation analysis.
Results with Fisher score >= 7 and Z-score >= 10 shown

TFBS	JASPAR ID	Z-score	Fisher score
Tal1::Gata1	MA0140.1	16.629	10.813

Supplemental Table 7f: Module 17 transcription factor binding site (TFBS) overrepresentation analysis.
Results with Fisher score >= 7 and Z-score >= 10 shown

TFBS	JASPAR ID	Z-score	Fisher score
ELK1	MA0028.1	17.646	10.238
INSM1	MA0155.1	14.577	9.455
Zfx	MA0146.1	13.328	8.785
Klf4	MA0039.2	20.059	7.999

Supplemental Data 8a: Conservation of Modules across Human Data Sets. Zsummary < 2 implies no evidence for module preservation, 2 < Zsummary < 10 implies weak evidence of preservation and Zsummary >10 implies strong evidence for module preservation.

Module	Module Size	Z Summary
0	1000	0.354
1	539	27.1
2	502	26
3	398	8.64
4	267	4.32
5	88	1.94
6	110	6.81
7	60	1.73
8	65	4.35
9	37	2.25
10	63	8.98
11	47	6.08
12	37	2.14
13	36	0.633
14	29	2.88
15	33	2.63
16	7	0.796
17	33	0.934

Supplemental Data 8b: Conservation of Modules across Species (human and procine Data Sets). Zsummary < 2 implies no evidence for module preservation, 2 < Zsummary < 10 implies weak evidence of preservation and Zsummary >10 implies strong evidence for module preservation.

Module	Module size	Z Summary
0	1000	1.84
1	539	14.8
2	502	5.57
3	398	9.47
4	267	7.68
5	88	3.05
6	110	3.11
7	60	0.445
8	65	3.4
9	37	5.43
10	63	6.14
11	47	6.27
12	37	4.35
13	36	-0.512
14	29	1.1
15	33	3.52
16	7	0.868
17	33	3.71

Supplemental Data 8c: Association of module eigengene with disease status. Human sepsis with multi-organ dysfunction vs controls

Module	Coefficient	P-value (FDR adjusted)
0	0.380067967	1.37E-06
1	-0.370028149	4.52E-06
2	0.378967138	1.58E-06
3	-0.314635576	0.000409186
4	-0.367401006	6.02E-06
5	-0.361177786	1.14E-05
6	0.375370487	2.45E-06
7	0.301751322	0.000858479
8	0.34711512	4.07E-05
9	0.362842149	9.64E-06
10	0.390928695	3.00E-07
11	-0.131472079	0.198079441
12	-0.096027896	0.352299397
13	0.361703691	1.08E-05
14	0.352482276	2.56E-05
15	0.395542961	1.43E-07
16	-0.232150298	0.016489467
17	-0.308515369	0.000587722

Supplemental Data 8d: Association of module eigengene values with disease status. Porcine model of ICUAW Day 5 sepsis vs Day 1 control

Module	Coefficient	P-value (FDR adjusted)
0	0.342010933	0
1	-0.343470633	2.22E-15
2	0.362021015	0
3	-0.238452868	0.004122182
4	-0.320736172	9.99E-16
5	-0.30230668	1.75E-08
6	0.163683402	0.036778161
7	-0.272556578	5.30E-06
8	0.424086818	0
9	0.427822514	0
10	0.358892396	0
11	0.042444773	0.697227349
12	-0.231994183	0.018551701
13	-0.381391573	0
14	0.327734961	0
15	0.301133419	7.08E-09
16	-0.022019174	0.809482794
17	-0.224111232	0.002619171
